# Unexplored Antarctic meteorite collection sites revealed through machine learning

**DOI:** 10.1126/sciadv.abj8138

**Published:** 2022-01-26

**Authors:** Veronica Tollenaar, Harry Zekollari, Stef Lhermitte, David M.J. Tax, Vinciane Debaille, Steven Goderis, Philippe Claeys, Frank Pattyn

**Affiliations:** 1 Laboratoire de Glaciologie, Université libre de Bruxelles, Brussels, Belgium.; 2 Department of Geoscience and Remote Sensing, Delft University of Technology, Delft, Netherlands.; 3 Pattern Recognition Laboratory, Delft University of Technology, Delft, Netherlands.; 4 Laboratoire G-Time, Université libre de Bruxelles, Brussels, Belgium.; 5 Analytical, Environmental, and Geo-Chemistry, Vrije Universiteit Brussel, Brussels, Belgium.

## Abstract

Meteorites provide a unique view into the origin and evolution of the Solar System. Antarctica is the most productive region for recovering meteorites, where these extraterrestrial rocks concentrate at meteorite stranding zones. To date, meteorite-bearing blue ice areas are mostly identified by serendipity and through costly reconnaissance missions. Here, we identify meteorite-rich areas by combining state-of-the-art datasets in a machine learning algorithm and provide continent-wide estimates of the probability to find meteorites at any given location. The resulting set of ca. 600 meteorite stranding zones, with an estimated accuracy of over 80%, reveals the existence of unexplored zones, some of which are located close to research stations. Our analyses suggest that less than 15% of all meteorites at the surface of the Antarctic ice sheet have been recovered to date. The data-driven approach will greatly facilitate the quest to collect the remaining meteorites in a coordinated and cost-effective manner.

## Teaser

Analyzing remote sensing data advances the search for Antarctic meteorites, revealing the ice sheet’s untapped potential.

## INTRODUCTION

Meteorites are parts of planetary bodies that formed and evolved throughout the evolution of the Solar System. These extraterrestrial rocks fell on Earth after surviving the passage through the atmosphere. Being directly accessible at the Earth’s surface, meteorites provide important insight into nebular and planetary processes. Antarctic meteorites are especially important in this context because of their pristine states and the existing legal framework that ensures their availability for scientific research (*1*).

**Fig. 1.**
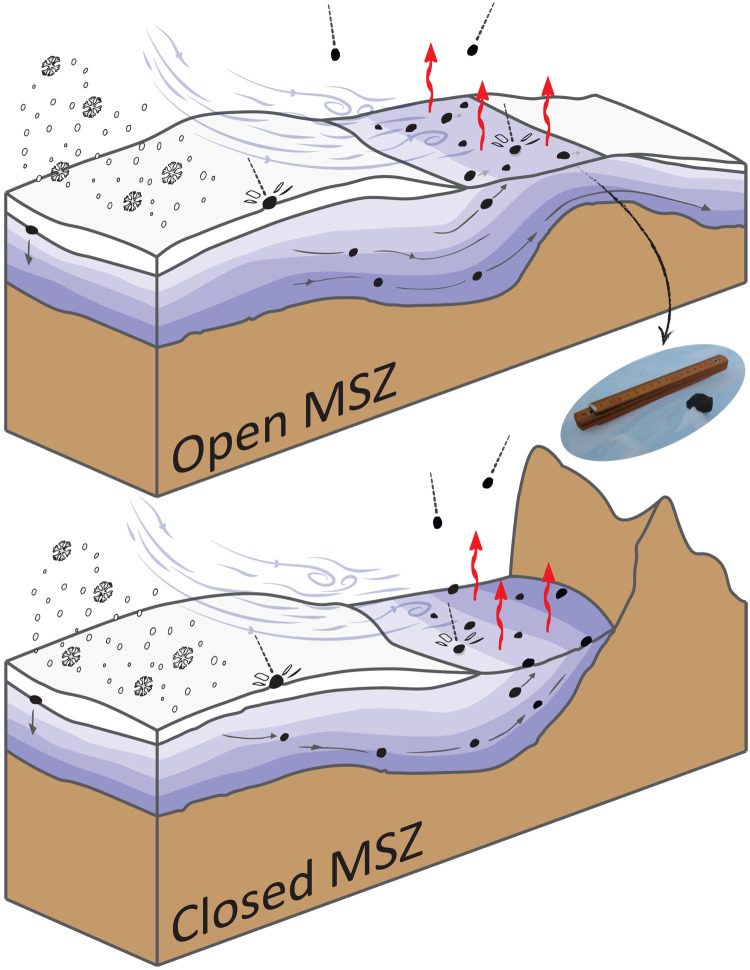
Schematic representation of two possible settings of the meteorite concentration mechanism, related to a submerged barrier (open MSZ) and to an emerged barrier (closed MSZ); not to scale. Bedrock (both subglacial and exposed) is shown in brown. Blue colors represent the ice (the darker, the older), and white represents snow. Accumulating snow is displayed, as well as the direction of katabatic winds, which enhance the ablation. The red arrows indicate the occurrence of ablation (sublimation). Gray arrows display the flow of the ice in which meteorites are embedded. Black dots represent meteorites. The picture of a meteorite and a folding rule for scale was taken during the JARE-54 (Japanese Antarctic Research Expedition)/BELARE (Belgian Antarctic Research Expedition) 2012–2013 expedition to the Nansen blue ice field (*60*).

When meteorites fall on the surface of the Antarctic ice sheet, they typically become entrapped in the ice sheet’s snow-covered accumulation area, which spans 98% of the continent (*2*, *3*). During the process in which snow accumulates, compacts, and transforms to ice, meteorites become embedded in the ice sheet (Fig. 1). These meteorites are then transported along with the ice that flows under gravitational forces toward the margins of the continent. Although most of the englacially transported meteorites end up in the ocean, a small fraction is brought back to the surface of the ice sheet in some of the continent’s blue ice areas (BIAs) (*4*). In BIAs, the annual ablation exceeds the accumulation (*2*, *5*). If the ice within a BIA contains meteorites, these meteorites eventually become exposed through the removal of the ice by ablative processes (sublimation). Moreover, the absence of snow accumulation in a BIA implies that meteorites falling directly on a BIA can remain exposed at the surface. Thus, if the flow of the ice and specific geographical and climatological settings combine favorably, a BIA can act as a meteorite stranding zone (MSZ) (Fig. 1). In MSZs, meteorites are concentrated at the surface, where they can be easily recovered during field missions, as, thanks to their color, they contrast with the underlying blue ice. These MSZs make Antarctica the most productive region for collecting meteorites on Earth; to date, about 62% of all meteorites recovered on Earth originate from Antarctica (*6*).

A conceptual model for the mechanism behind MSZs was first proposed in the early 1970s, after a Japanese expedition found nine meteorites at a BIA in 1969 (*7*, *8*). Subsequently, many searches for meteorites were conducted, and the mechanism behind MSZs was studied at multiple individual ice fields (*9*–*11*). The concentrating mechanism has been generalized qualitatively, describing the various influencing factors, as well as the different settings in which meteorites are concentrated (*4*, *12*). For example, when the ice flow meets a submerged barrier (open MSZ) (Fig. 1) or an emerged barrier (closed MSZ) (Fig. 1), the flow is slowed down and redirected toward the surface due to a buttressing effect (*4*, *5*, *12*, *13*). The contribution of driving factors behind meteorite concentrations (e.g., direct infall, ablation, or ice flow) differs for individual MSZs. In MSZs, meteorites stay at the surface for up to thousands of years (*9*, *14*), during which they can be transported by the almost stagnant ice flow (and potentially wind), until they eventually reach the edge of the MSZ and (re-)enter the ice sheet.

Many of today’s known MSZs were discovered coincidentally, and to date, the identification of new MSZs remains a very labor-intensive process that strongly relies on chance and past experience. Potential MSZs are typically identified through visual examination of remote sensing data of BIAs and their vicinity, after which candidate MSZs are visited by snowmobile or helicopter, to investigate whether a meteorite concentration is present (*15*). The discovery of meteorite concentrations thus partly depends on the expertise and experience of the persons examining maps and imagery, and largely on costly field reconnaissance visits. Because of this big human factor in the reconnaissance approach, it is most likely that major MSZs are still to be discovered.

Here, we combine the wealth of recent remote sensing observations and derived products over the Antarctic ice sheet with machine learning techniques to perform a continent-wide systematic analysis toward the detection of MSZs. A first attempt toward predicting areas containing concentrations of meteorites was recently performed by Evatt *et al.* (*14*). With a physics-based approach, they calculated the spatial flux of meteorite falls using data of 13 systematically searched Antarctic MSZs. Through inversion, they estimated physical parameters and used these to derive the density of meteorites for a given area (*14*). However, when using this approach at the continental scale, considerable uncertainties are introduced due to the lack of information on small-scale processes. For instance, wind speed, which is essential when estimating ablation, is not well constrained for most BIAs as regional climate models fail to resolve the complex surface settings and topography at MSZs (*16*). Moreover, complex interactions that can make meteorites (in)accessible are poorly represented in such an approach. Higher temperatures, for instance, not only lead to increased ablation (enhancing surficial meteorite concentration) but also result in increased weathering and potential sinking of meteorites into the ice (reducing surficial meteorite concentration). To overcome these limitations, we introduce a data-driven method, where multiple datasets with quantifiable uncertainties are used to predict the probability to find meteorites given an observation anywhere in Antarctica. This probability cannot be obtained from a simple overlay analysis, as individual MSZs exhibit different glaciological and geographical characteristics (*12*), implying that correlations between the datasets need to be considered. Therefore, we use a machine learning algorithm in which a classifier is trained through positive and unlabeled learning (*17*). This approach does not include negative observations (areas that are reported to be absent of meteorite concentrations) as a part of the training process, as many of these observations have a limited reliability and are not representative for all locations absent of meteorites. Instead, negative observations are used for the calibration (feature selection) and the evaluation of the classification. Through this approach, we generate and extensively evaluate the continent-wide prediction of where to find meteorites in Antarctica. With these predictions, we define a “where-to-go” index as a new and valuable tool for expensive meteorite recovery programs that allows prioritizing reconnaissance of BIAs with a high potential.

## RESULTS

### Observations

As meteorites are always recovered from BIAs (except in some very rare cases) (*18*), we only consider these areas and their immediate surroundings, based on a recent BIA dataset (*3*, *19*, *20*). The BIAs (including a 1-km expansion, see Materials and Methods) are overlaid with a regular spaced grid of 450-m resolution, aligned with the grid of Antarctic-wide surface velocity data (*21*). The resolution is chosen according to the availability of the data, and although it is not fine enough to fully capture the inter-BIA (re)distribution of meteorites, it allows a continent-wide intercomparison of the potential of BIAs to contain meteorites. Moreover, the machine learning model is tested for robustness by purposeful elimination of data to understand the sensitivity to the scale of the variables with respect to the arbitrary resolution (see Discussion). Grid cells containing one or multiple meteorite finds are labeled as positive observations, while all other cells are considered unlabeled observations, except for 8726 observations that are reported to be without meteorites and that are used for the calibration of the classifier (i.e., negative observations; fig. S1). The resulting training dataset consists of 2554 positive observations and 2.1 million unlabeled observations.

### Feature definition

**Fig. 2.**
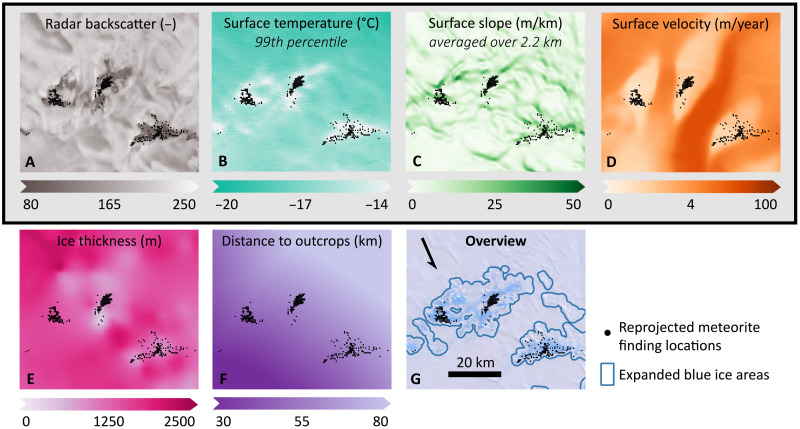
Overview of the features in the area of Elephant Moraine (76^°^17′S, 157^°^20′E). The reprojected meteorite finding locations are shown in all panels (black dots), while the expanded BIA outlines are shown in the lower right panel. The four features framed with a black line are used for the final classification. (**A**) For the radar backscatter (200-m resolution), the dataset RAMP AMM-1 SAR Image Mosaic of Antarctica v2 (*22*) is used. No unit is indicated, as the values of the dataset represent the radar backscatter intensity in eight-bit digital numbers (*22*). (**B**) For the 99th percentile of the surface temperature (1000-m resolution), preprocessed observations of MODIS, from 1 January 2001 to 1 January 2020, are used (MOD11A2 MODIS/Terra Land Surface Temperature Daytime, 8-Day Global, V006) (*29*). (**C**) The surface slope over 2.2 km is calculated using the Reference Elevation Model of Antarctica (*33*) at 200-m resolution, and a dataset of rock outcrops (Rock Outcrop medium resolution v7.1) (*34*, *40*). (**D**) For the surface velocity (450-m resolution), MEaSUREs Phase-Based Antarctica Ice Velocity Map v1 (*21*) is used. (**E**) For the ice thickness (500-m resolution), MEaSUREs BedMachine Antarctica v2 (*36*) is used. (**F**) For the distance to outcrops, the dataset of rock outcrops (*34*, *40*) is used. (**G**) The background image in the lower right overview is taken from the Center-Filled Landsat Image Mosaic of Antarctica (LIMA) Project (*61*).

MSZs are generally known to (i) expose blue ice with (ii) cold surface conditions resulting in surface mass loss through sublimation (rather than surface melt) and to be characterized by (iii) very low ice flow velocities due to the presence of (sub)surface barriers (*4*, *12*). These three main characteristics are described through six features with a continent-wide coverage (Fig. 2).

The presence of blue ice is assessed through radar backscatter data (*22*), as backscatter can be used to exclude snow-covered and rock-exposed areas in the vicinity of BIA outlines [that are included due to the 1-km expansion and/or are erroneously marked as blue ice in the original dataset (*3*), see Materials and Methods]. As radar signals penetrate into thin snow layers (*23*), occasional snow covering of blue ice (*24*) has a limited influence on the intensity of the radar backscatter.

Direct estimates of the components of the surface mass balance (e.g., accumulation, melt, and sublimation) are not available over the entire continent on a spatial resolution that is sufficient to capture local processes at MSZs. MSZs have typical sizes of tens of square kilometers and are characterized by topographic complex terrain, while modeled surface mass balance products (e.g., from climate models) and measurements are only available on relatively coarse resolutions (*16*, *25*). Therefore, we rely on two indirect quantities related to the surface mass balance with a continent-wide availability of sufficient resolution: the surface temperature and the surface slope.

Higher surface temperatures at MSZs lead to an increased probability of surface melt, resulting in enhanced weathering of meteorites (*12*, *26*). Moreover, when temperatures are high, the additional radiation absorbed by meteorites, compared to the surrounding ice (*27*), makes meteorites prone to melt the underlying ice, causing them to sink (*12*, *28*). The two unfavorable processes (surface melt and sinking of meteorites) peak during extreme heat events. However, the extremity of the heat events is unknown, and therefore, various percentiles of the 2001–2020 distribution of eight-daily mean surface temperatures [derived from Moderate Resolution Imaging Spectroradiometer (MODIS) satellite data] (*29*) are considered (70th, 90th, 95th, and 99th).

Increasing surface slopes are known to accelerate katabatic winds (*30*, *31*). The strength of these katabatic winds is related to the mass removal at the surface of BIAs through sublimation and snow drift (*5*, *31*, *32*). Since the scale over which the surface slope influences local wind speeds is uncertain and location dependent, the surface slope of the ice sheet has been calculated over various distances (0.4, 2.2, and 5 km) by masking and filtering surface elevation data (see Materials and Methods) (*33*, *34*). Moreover, as the ice flow is directly related to the surface slope, this feature is also a proxy for the local ice flow.

The very limited ice flow, characteristic for MSZs, is represented by the magnitude of the surface velocity. The surface velocity is taken from the MEaSUREs (Making Earth System Data Records for Use in Research Environments) Phase-Based Antarctica Ice Velocity Map (*21*), which relies on InSAR (Interferometric Synthetic Aperture Radar) techniques for slow-flowing areas and feature and speckle tracking for fast-flowing areas (*35*). Other features derived from the surface velocity (e.g., divergence, curl, and change of ice thickness along the flow line) were investigated but discarded, as even small errors in the direction of the low surface velocities strongly influence their derivatives. The low velocities are linked to the presence of (sub)surface barriers, which play an important role in diverting meteorites to the surface (Fig. 1). For describing the subglacial barriers, multiple ice thickness datasets are available (*36*–*38*). All these datasets are subject to uncertainties with a spatial component that is strongly related to the spatial coverage of ice thickness measurements. Here, we use the recent subglacial bed topography data from BedMachine Antarctica (*39*). Last, to represent surface barriers, a feature that represents the distance to the nearest outcrop is calculated using a dataset of outlines of exposed bedrock (*34*, *40*).

### Feature selection

**Fig. 3.**
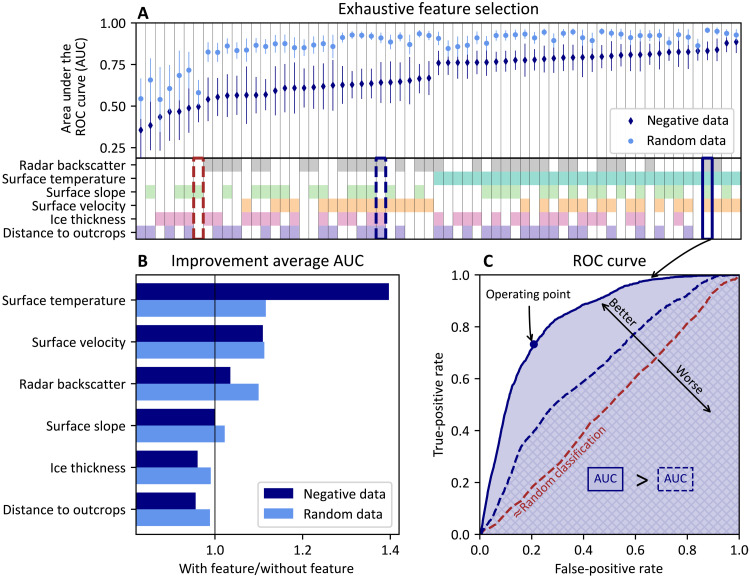
Exhaustive feature selection. (**A**) The (average) AUC is calculated for all 63 combinations of the six features. Two sets of calibration data are used, one with actual negative data (based on which the results are arranged; dark blue) and the other with a random selection of the unlabeled data (light blue). One SD of the AUCs (obtained in the cross-validation) is shown as error bar. (**B**) Improvement of the classifier when including a given feature. The mean AUC of the 32 combinations with a certain feature is divided by the mean AUC of the 31 combinations without a certain feature. (**C**) Three ROC curves (obtained with negative data) illustrating the relation between the false-positive rate and the true-positive rate. These rates are estimated by applying the classifier to calibration data and comparing the predicted class (positive or negative) to the actual class. By varying parameters of the classifier (see Materials and Methods), different true-positive and false-positive rates are obtained. To compare ROC curves, the area under the curve (AUC) is used. In this example, the AUC when using the four selected features (radar backscatter, surface temperature, surface slope, and surface velocity; solid line) is larger than the AUC with a different combination of features (radar backscatter, surface velocity, ice thickness, distance to outcrops; blue dashed line). When solely relying on the surface slope (red dashed line), the classification approximates a random classification, as the classifier is not able to distinguish positive from negative observations.

The influence of the six features on the classifier is evaluated using two average receiver operating characteristic (ROC) curves (Fig. 3C) obtained in a cross-validation procedure (see Materials and Methods). The ROC curve shows the relation between the true-positive and the false-positive rate, estimated with calibration data consisting of positive and negative observations. In this case, two different sets of negative calibration data are used to construct two ROC curves. (i) The first set consists of the actual negative observations deduced from fieldwork reports (negative data; fig. S1). The resulting ROC curve reflects the capability of the classifier to distinguish positive observations (i.e., areas where meteorites have been found) from areas that have been selected and visited by experts but turned out to be absent of meteorites. (ii) The other set of calibration data consists of 9000 randomly selected unlabeled observations (random data). This second set of calibration data is needed, as the actual negative observations in the first set of calibration data form a biased sample of all negative observations. Areas within the expanded BIAs that have never been searched, as they obviously do not contain meteorite concentrations (e.g., exposed bedrock or snow-covered areas adjacent to blue ice), are not represented in the negative observations. Therefore, the first ROC curve (negative data; Fig. 3) does not reflect potential biases of the classifier toward obviously meteorite-free areas. To avoid this, we randomly sampled unlabeled observations to construct this complementary ROC curve that reflects the capability of the classifier to distinguish positive observations from arbitrary areas within the expanded BIAs.

Features are selected by comparing the performance of the classifier with a given feature to the performance without that feature (Fig. 3B), by relying on an exhaustive feature selection in which all possible combinations of features are considered (Fig. 3A). The performance of the classifier is expressed as the (average) area under the ROC curve (AUC) (Fig. 3C). For four of the six features, the AUC increases when these are included (compared to the case where they are omitted): surface temperature, surface velocity, radar backscatter, and surface slope (Fig. 3B).

**Fig. 4.**
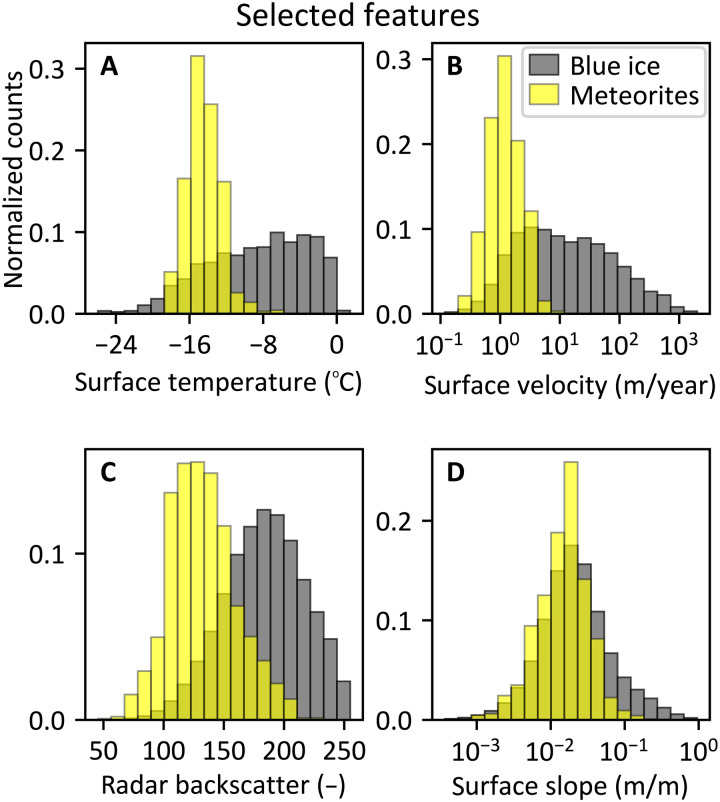
Histograms of the four selected features. (**A**) Surface temperature (99th percentile), (**B**) surface velocity, (**C**) radar backscatter, and (**D**) surface slope (averaged over 2.2 km). Values for the ca. 2.1 million unlabeled observations at the expanded BIAs are in gray (“blue ice”) and the 2554 positive observations are in yellow (“meteorites”). Histograms of other features are provided in the Supplementary Materials (fig. S4). For references and details about the datasets used, refer to the caption of Fig. 2.

The surface temperature and the surface velocity are the two most important features; when they are not included, the AUC reduces sharply (Fig. 3B). These two features allow distinguishing positive observations from places that were judged as potential MSZs by experts but turned out to be absent of meteorites (i.e., negative observations); as with these features, the AUC with negative data is the largest (Fig. 3A). For the surface temperatures, the largest AUCs are obtained when considering the higher percentiles (90th, 95th, and 99th) of the 19 years of eight-daily surface temperatures (*29*), while the AUC is strongly reduced when considering the 70th percentile (fig. S2). The enhanced performance for the high percentiles is particularly pronounced for the Grove Mountains and Frontier Mountain, where using the 70th percentile for the temperature reduces the area under these individual ROC curves to 74 and 77%, respectively (of the AUC of the 99th percentile, fig. S2). Over MSZs, only 1% of all temperature observations during the 19-year period exceeds −9.01°C (99th percentile; Fig. 4), which is very close to the threshold of −10°C suggested for the loss of meteorites due to sinking (*12*). The ice flow velocities confirm that meteorites are always found in areas with limited flow, with values up to 6.07 m/year (99th percentile; Fig. 4).

When including the radar backscatter and the surface slope, the performance of the classifier also improves (Fig. 3B). These two features allow discriminating between positive observations and random places within the expanded BIAs, such as snow-covered areas and rock-exposed areas, as adding these two features leads to the largest AUC with random data (Fig. 3A). The radar backscatter distinguishes exposed blue ice from snow-covered areas and areas where melt and refreezing take place (see Materials and Methods for an interpretation of the values) (*41*). Surface slopes at MSZs do not differ greatly from surface slopes of unlabeled observations (Fig. 4; see also the nearly random classification based solely on the surface slope in Fig. 3C). Nevertheless, the surface slope appears in the feature selection (Fig. 3B), confirming that there is a relevant interplay between other features and the surface slope. This interplay pleads for our machine learning approach instead of a simple overlay analysis. The distance over which the surface slope is calculated (0.4, 2.2, or 5 km) has a very limited influence on the average performance of the classifier (fig. S3). Surface slopes (averaged over 2.2 km) at MSZs have values up to 104.2 m/km (99th percentile; Fig. 4).

The ice thickness and the distance to outcrops (moderately correlated, *R* = 0.63) do not improve the performance of the classifier, either because the features are not important for predicting the presence of a meteorite concentration or because biases in these datasets are too large for our application. For the final classification, the surface temperature (99th percentile), surface velocity, radar backscatter, and surface slope (averaged over 2.2 km) are used. The close connection between the four selected features (Fig. 2) and the current understanding of the concentration mechanism allows for a meaningful interpretation of the obtained classification, which is important given the limited quality and restricted availability of labeled observations.

### Classification

**Fig. 5.**
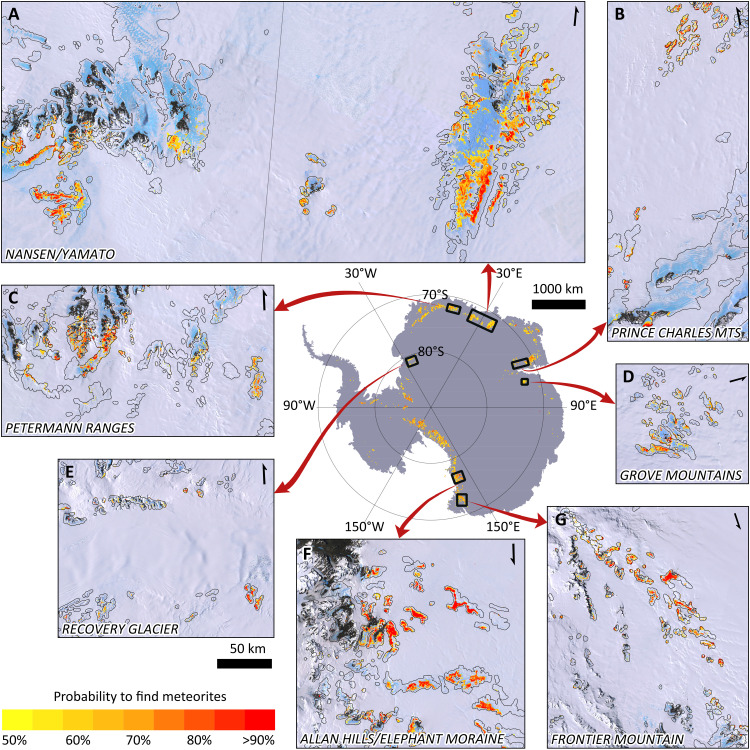
Antarctic meteorite hotspot map with positive classified observations. In the central overview map, the size of the positive classified observations is exaggerated for visual contrast, while in the submaps (**A** to **G**), the positive classified observations are shown to scale. The expanded BIAs over which the classification is performed are delineated in black. The “probability to find meteorites” at a given location corresponds to the a posteriori probability (see Eq. 4.1 in Materials and Methods).

In total, 106,687 observations are classified as positive (Fig. 5). This classification is evaluated with independent test data (i.e., not used for training the classifier), both (i) locally (at 450-m grid-cell level) and (ii) at the MSZ level.

(i) At the grid-cell level, positive and negative observations on the 450-m grid (fig. S1) are used as test data. The ratio of positive to negative observations in the test data (approximately 1:4) is closely reproduced in the classification (see confusion matrix, table S2). With an accuracy of 78% (Table 1), the classifier can determine whether an observation is positive or not. For the classifier, it is relatively easy to eliminate grid cells where no meteorites are found: With a specificity of 86%, only a small fraction of the negative observations is misclassified. However, the estimated precision of 47% suggests that there is a considerable chance of not finding any meteorite in a positively classified 450-m grid cell (Fig. 5). When considering data representative for the entire spectrum of areas absent of meteorites (i.e., a random selection of the unlabeled data, versus the negative data consisting of nonproductive areas visited during reconnaissance missions), there are fewer false positives, resulting in an improved precision of 66%. Not all positive grid cells are identified by the classifier, as indicated by the sensitivity of 48%. The estimated probabilities (Fig. 5) reveal spatial patterns within groups of neighboring positive classified grid cells. These patterns need to be interpreted with care, as these variations can be simply caused by uncertainties and/or resampling of the data used for the classification, which is confirmed by the relatively low precision and sensitivity. Hence, the predictions on the presence of meteorites per grid cell are not ideal for the detailed planning of meteorite recovery missions (i.e., where to go exactly within an MSZ).

**Table 1.** Performance metrics of the classification. In the column “Equation,” TP denotes the number of true positives, FP indicates the number of false positives, FN denotes the number of false negatives, and TN indicates the number of true negatives. The column “Grid-cell level” gives the results of the evaluation using an independent test dataset (i.e., not used for training) consisting of positive and negative observations on the 450-m grid. The column “MSZ level” provides the results of the evaluation of the postprocessed results (i.e., MSZ outlines) using a test dataset consisting of known MSZs and non-MSZs.

**Metric**	**Equation**	**Grid-cell level**	**MSZ level**
Precision	$\frac{\text{TP}}{\text{TP}+\text{FP}}$	47.3%	88.6%
Sensitivity	$\frac{\text{TP}}{\text{TP}+\text{FN}}$	48.2%	81.6%
Specificity	$\frac{\text{TN}}{\text{TN}+\text{FP}}$	86.2%	84.6%
Accuracy	$\frac{\text{TP}+\text{TN}}{\text{TP}+\text{TN}+\text{FP}+\text{FN}}$	78.4%	82.8%

(ii) For an alternative evaluation, at the MSZ level, the positive classified observations are clustered to obtain outlines of MSZs. Here, isolated positive and negative pixels are eliminated by applying a majority filter (see Materials and Methods), and MSZs smaller than 4 km^2^ are ignored, resulting in a dataset of 613 MSZs. In the evaluation, the identified MSZs are compared to known MSZs and non-MSZs that have not been used in the training process (except for eight positive grid cells; see table S3 for details). Some of the MSZs are only partially classified, and these MSZs are conservatively counted as 0.5 instead of 1. For the non-MSZs, the percentage of positive classified pixels within the area is calculated, after which a threshold of 20% is set (i.e., if more than 20% of the non-MSZ is misclassified as MSZ, it is considered a false positive). Generally, the evaluation at the MSZ level reveals that the classifier is very successful at classifying both MSZs and non-MSZs (Table 1). The precision at the MSZ level, which is crucial to avoid costly reconnaissance missions to misclassified non-MSZs (false positives), is 88.6%. Normally, a high precision comes at the cost of a low sensitivity, but the estimated sensitivity is also relatively high with 81.6%, indicating that the classifier correctly identifies most of the existing MSZs. The specificity reveals that 84.6% of the non-MSZs are correctly classified as non-MSZs. In the hypothetical situation that previous meteorite-search expeditions would have had our classification at hand, this high specificity indicates that most of the areas absent of meteorites would not have been visited. Moreover, the two non-MSZs partially classified as MSZ (table S3) could be considered for a revisit, as the misclassification might be attributed to possible temporal circumstances that prevented a proper assessment in the field (e.g., considerable snow cover). Nearly 83% of the known MSZs and non-MSZs used in the evaluation are accurately classified. Hence, the presented classification is very valuable for reconnaissance missions, i.e., to determine the presence of meteorites at potential MSZs.

## DISCUSSION

The independent evaluation suggests that our encompassing and automated data-driven approach in identifying MSZs is an improvement compared to the current manual approaches that are largely based on expert knowledge (see Introduction). Moreover, the classifier captures relevant interacting phenomena by accounting for the interplay between the four selected features (surface temperature, surface velocity, radar backscatter, and surface slope), which cannot be achieved by a simple (manual or automatic) overlay analysis. For example, at the blue ice fields at Martin Hills, visited in 1998 and in 2007 (*42*, *43*), maximum temperatures are low (−14°C), ice flow is slow (4.4 m/year), blue ice is present, and surface slopes are relatively gentle (31 m/km). Yet, no meteorites are found, as correctly predicted by the classifier. In some other areas, the absence or presence of meteorites can be explained by considering the values of the individual features. For example, at the blue ice fields near Morris Cliff, visited in the 1997–1998 field season (*44*), no meteorites are found, because, on occasional days, the temperature is too high (up to −7.7°C). Another example is the bare ice field south of Mount Bamse, visited in 1987 (*45*), where no meteorites are found because the ice flow is too fast (16 m/year). The capacity of the classifier to distinguish these non–meteorite-bearing places from potential MSZs over the entire Antarctic continent constitutes a major improvement in the planning of costly reconnaissance missions.

The performance of the classifier is not largely influenced by changes in the positive training data, which confirms that the classifier is applicable to the real-world, noisy data. Four scenarios with alternative sets of positive observations have been investigated (fig. S5). (i) Leaving out all observations of any of the nine most productive field sites. This data reduction does not affect the classification, indicating that none of the field sites is essential for the training data. (ii) Using the original 12,906 observations, which contrasts with our standard approach in which meteorites are reprojected on a regular grid to 2554 locations. Using the original meteorite finding locations slightly reduces the performance of the classifier, as many observations are then correlated. (iii) Ignoring isolated finds by discarding grid cells with only a single meteorite find, which also (unintentionally) eliminates more widely spaced meteorite finds. This selection reduces the number of positive observations available for training from 2554 to 1359 and does not affect the performance of the classification. (iv) Discarding meteorites lighter than a certain threshold, to reduce the effects (noise in positive training data) related to the wind-driven transport of (light) meteorites on the surface. As the threshold for wind displacement is strongly influenced by local circumstances (*12*, *46*, *47*), three thresholds are investigated: 100, 150, and 200 g, resulting in 1179, 975, and 825 observations, respectively. In all three cases, the performance of the classifier does not change, indicating that wind displaced meteorites do not influence the classifier and/or that there are few observations of meteorites displaced over larger distances (with respect to the applied resolution of 450 m). In summary, with a maximum reduction of the AUC by 5% (in case the nonreprojected finding locations are used), the performance of the classifier is not very sensitive to perturbations of the training data.

Future improvements of the MSZ prediction depend on the quality and availability of observations. The classifier tends toward underfitting (AUC of the test data is 97 to 98% of the AUC of the calibration data, fig. S6), indicating that additional features are likely to improve the performance of the classifier even further. Moreover, regarding the set of positive observations, only a third of more than 45,000 Antarctic meteorite finds (*6*) has been used for the classification, as no detailed location data are available for the other meteorites. Given the uncertainties of the negative observations and (to a lesser degree) the positive observations, a relatively simple, statistics-based machine learning algorithm is used here. With increasingly reliable observations, advanced techniques, such as deep neural networks, could improve the classification. However, this improved classification will then likely come at the cost of a more difficult physical interpretation of the results (i.e., “more of a black box”) (*48*). In future work, an insight into the distribution of meteorites within a MSZ will be of large value to reduce the high costs related to extensive grid searches over entire MSZs. To obtain reliable intra-MSZ predictions, a more local approach is needed, given the wide range of local settings of MSZs. For this, recent developments in the field of remote sensing are highly promising, such as the emergence of high-resolution datasets with low uncertainties.

**Fig. 6.**
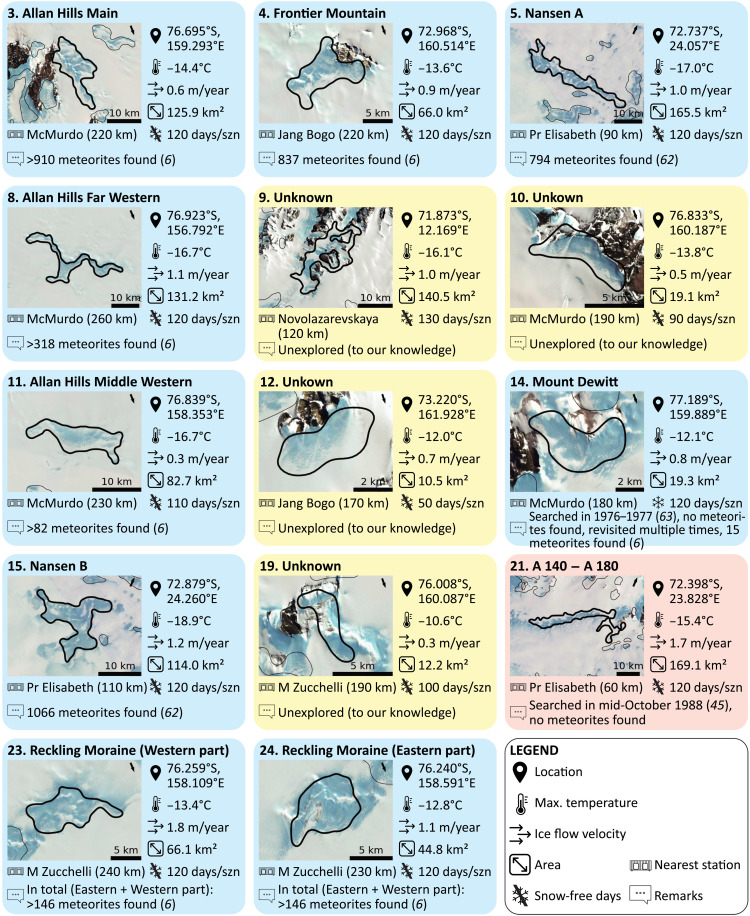
MSZs with a high potential. MSZs larger than 10 km^2^ with a high expected feasibility and success of a field visit. The panel titles give the name of each MSZ and its ranking based on the where-to-go index (“1” being the highest). The panel colors and remarks indicate whether the MSZ has been successfully visited (meteorites predicted and found in the field, blue), unexplored (containing meteorites but not visited yet, yellow), or unsuccessfully visited (meteorites predicted but not found in the field, red). Background images are false-color, pan-sharpened images of the LIMA project (*61*). The maximum temperature (99th percentile) and the ice flow velocity represent the median value of the observations within the MSZ (see Materials and Methods). “Snow-free days” represents the number of days per field season (szn) (November to February) that at least 50% of the MSZ (or for MSZs larger than 20 km^2^, at least 10 km^2^) is snow-free (see Materials and Methods).

The classified 613 MSZs distributed over the entire Antarctic continent correspond to both known and unexplored MSZs. The fact that the classification successfully identifies over 80% of the known MSZs gives us a high confidence in the classifier’s prediction of unexplored MSZs. To directly use the classification as a tool for planning future missions, we established a “where-to-go index” that ranks each MSZ according to quantities that roughly reflect the expected feasibility and success of a field visit. To obtain the where-to-go index, the MSZs are first ordered three times according to three different parameters: (i) the distance to the nearest Antarctic research station, (ii) the median of the a posteriori probability of the grid cells within the (potential) MSZ, and (iii) the presence of temporary snow layers (see Materials and Methods). The summation of the three rankings forms the where-to-go index. A table of all ranked MSZs, providing the location, the maximum temperature, the ice flow velocity, an indication of the number of snow-free days per season, the area, and the (distance to the) nearest research station, is provided in the Supplementary Materials (table S6; “Data and materials availability”). An analysis of the highest ranked MSZs larger than 10 km^2^ indicates that several of these MSZs were already visited (Fig. 6). Other highly ranked sites were visited, yet no meteorites were found, partly illustrating the limitations of either the classifier or the negative data (i.e., meteorites may have been present, but not detected, for instance, due to temporary snow patches). Last, some of the MSZs with a high rank have (to our knowledge) not been visited to date, illustrating that the unparalleled potential of Antarctica to yield meteorites has not been fully explored yet.

By combining estimates on (i) the total of true-positive and false-negative observations (based on the number of positive classified observations, 106,687), (ii) the precision (47 to 81%) (Table 1 and table S1), (iii) the sensitivity (48 to 74%) (Table 1 and table S1), and (iv) the average number of meteorites per positive observation (12,906/2554 ≈ 5 meteorite finds per pixel, where pairing of meteorites is not considered, see Materials and Methods), we predict that 340,000 to 900,000 meteorites are present at the surface of the ice sheet. This implies that only a small fraction (5 to 13%) of all meteorites has been recovered from the Antarctic ice sheet to date. Therefore, future data-driven meteorite recovery missions will allow collecting a large number of meteorites remaining on the ice sheet (among which we expect several rare types, such as angrites, brachinitres, or Martian meteorites, see fig. S7). Collecting this unique and well-preserved material will further enhance the understanding of our Solar System.

## MATERIALS AND METHODS

### Data for observations

For the unlabeled observations, a recent BIA dataset is used (*3*, *19*, *20*). Meteorites are (almost always) (*18*) found on blue ice; however, a substantial part (17%) of the meteorite finding locations appear to be outside the outlines of the BIA dataset. To include these observations and to account for uncertainties in the data, the BIA outlines are expanded with a 1-km buffer. In addition, in the Queen Fabiola Mountains, the BIA outlines have been manually adjusted to include all the blue ice from one of Antarctica’s prime meteorite collection locations: the Yamato BIA. BIAs with an average altitude below 200 m (calculated using the surface elevation data) (*33*) are not considered because meteorite concentrations there are highly unlikely (they are typically found above 1500 m, due to the adverse climatic conditions at lower elevation) (*49*).

For the positive observations, we use the data provided in the Meteoritical Bulletin Database (*6*), as consulted on 7 May 2019 (entries after this date are used for independent testing, details below). Of the 28,937 coordinates provided in this database, 14,128 are unique, and 12,906 are locations with a single meteorite. Thus, 1222 locations exist where multiple meteorites have the exact same coordinates (on average ca. 13 meteorites per location). Presumably, the actual coordinates of these meteorite finding locations are unknown, and therefore, these are approximated for all meteorites in a region with a single coordinate pair. Given the hereby induced uncertainties, these 1222 locations are not used. Of the remaining 12,906 locations with a single meteorite, 38 have no other meteorite locations within a radius of 4 km. These 38 locations may represent isolated meteorite finds (i.e., meteorite finds unrelated to a meteorite concentration) or observations at MSZs that have not yet been searched thoroughly.

Meteorites are named after the finding location of the meteorite, here referred to as the field site, and sometimes, the database also provides the name of the ice field. Given the long history and unexpected successes of meteorite searching campaigns, in some cases, the field sites refer to glaciologically and geographically unconnected areas, and they would likely be designated as distinct field sites nowadays, because of the large number of specimens collected in these areas (*50*). Despite these inconsistencies, we use the field sites as provided in the database for dividing the positive observations into 10 different sets in the cross-validation (see the “Classifier” section). Moreover, most entries in the database represent individual specimens, as only the most obviously fragmented proximal stones are usually paired as one single meteorite in the field (*50*). Despite the importance of considering pairing of meteorite specimens (*12*, *51*), to our knowledge, no systematic pairing campaigns on Antarctic meteorites have been performed (*50*, *52*, *53*), and pairing data are often not updated in the database. Therefore, the (reprojected) observations are used as provided in the database, notwithstanding the limitation this imposes on the results. Positive observations used for testing are gathered from fieldwork reports and the literature, consisting of maps showing the spatial distribution of meteorite finds (table S4). These maps are georeferenced using the software QGIS. Subsequently, the finds are reprojected according to the same procedure as the positive training data (see Results). The positive testing data are complemented with entries that have been added to the Meteoritical Bulletin Database (*6*) after 7 May 2019 (table S4).

The negative observations are deduced from fieldwork reports, which provided essential information on unsuccessful meteorite searches. From the descriptions in the literature, polygons have been drawn using Google Earth Pro (v. 7.3.2.5776), and these have been rasterized (see the “Observations” section). A table indicating the sites of the negative observations, including references, descriptions of the location, and information on the search, is provided in the Supplementary Materials (table S5). The negative observations are arbitrarily split up into a set of field sites used for calibration and a set of field sites for testing (42 and 13 field sites, respectively; table S5). Given the limited details on the circumstances and intensity of the searches, the validity of the absence of meteorites is in some cases questionable. Therefore, we always use the entire set of negative observations during calibration (in contrast to the positive observations that have been split up according to their field sites in the cross-validation procedure, see the “Classifier” section).

### Data for features

The radar backscatter at observation locations is obtained by linearly interpolating the 200-m-resolution data of RAMP (Radarsat Antarctic Mapping Project) AMM-1 (first Antarctic Mapping Mission) SAR (Synthetic aperture radar) Image Mosaic of Antarctica v2 (*22*). This continent-wide product contains integer grayscale values between 0 and 255, representing the radar backscatter intensity. Given the regular spacing of the observations, the linear interpolation results in duplicates (there are ca. 30,000 unique values, while ca. 2.1 million observations exist). Duplicates form a problem in the cross-validation of the bandwidth of the kernel density estimation (see the “Classifier” section), as they cause the smallest bandwidth to have the maximum log-likelihood. To resolve this, random Gaussian noise with an SD of 0.25 is added to the observations.

The surface temperature at observation locations is obtained by taking the 99th percentile (as well as the 70th, 90th, and 95th percentiles, see the “Feature definition” section) of all eight-daily surface temperature estimates obtained from 1 January 2001 to 1 January 2020 (MOD11A2 MODIS/Terra Land Surface Temperature Daytime, 8-Day Global, V006) (*29*) for the nearest neighbor of the observation. As the 1000-m-resolution data have been interpolated using the nearest neighbor method, duplicates exist. Therefore, random Gaussian noise with an SD of 0.04°C is added to the observations.

The surface slope at observation locations is obtained by linearly interpolating continent-wide surface slopes, which are calculated with the central difference on masked and filtered 200-m-resolution surface elevation data of the Reference Elevation Model of Antarctica (*33*). As the interest lies in the surface slope of the ice, outcropping bedrock is masked using a classification of outcrops provided by the Antarctic Digital Database (Rock Outcrop Medium Resolution v7.1) (*34*, *40*). Filtering is performed by applying an averaging filter with a circular-shaped footprint, to avoid sensitivity to the orientation of the axes. The diameter of the footprint is 2.2 km (equal to ca. five times the mean ice thickness at positive observations); in addition, a footprint of 5 km is investigated, as well as no filtering to obtain the averaged surface slope over 0.4 km. Because of errors in the outcrop outlines, the number of observations is reduced with 13 to 2541 when the diameter of the footprint equals 2.2 km. The observations are log-transformed to reduce the skewness of the distribution.

The surface velocity at observation locations is obtained by calculating the magnitude of the surface velocity from the directional components of the 450-m-resolution MEaSUREs Phase-Based Antarctica Ice Velocity Map v1 (*21*). The observations are log-transformed to reduce the skewness of the distribution.

The ice thickness at observation locations is obtained by linearly interpolating the original 500-m-resolution data of MEaSUREs BedMachine Antarctica v2 (*36*). The distance to outcrops at observation locations is obtained by linearly interpolating a 500-m-resolution rasterized approximation of the distance to outcrops. This approximation is made by considering buffers of increasing length (exponentially increasing from 400 to 1800 m in 750 steps) around a dataset of outcrop outlines (Rock Outcrop Medium Resolution v7.1) (*34*, *40*). This method results in duplicate observations, which are adjusted by adding random Gaussian noise with an SD of 0.1 km. To avoid negative values due to the additional noise for observations very close to exposed bedrock (<400 m), the absolute value of the noisy distance to outcrops is considered. The observations are then increased by 0.1 km and log-transformed to reduce the skewness of the distribution.

### Classifier

The positive and the unlabeled observations are used to train the classifier. The negative observations are only used for the calibration of the classifier, because of their limited reliability. Training a classifier with this nontraditional training set is referred to as positive and unlabeled learning (PUL) (*17*, *54*). PUL is successfully applied in classification problems occurring in real-world domains, such as land surface classifications using remote-sensing data (*54*) or classification of genes or proteins in molecular biology databases (*17*). As there is neither prior nor posterior motivation that the data are distributed normally, we use a kernel density estimation (KDE) to estimate the multidimensional density distributions of the positive and unlabeled observations (*55*), and we combine these probability density estimates with Bayes’ rule to get the final classifier. To briefly describe the underlying theory and the necessary adjustments due to the nontraditional training set of positive and unlabeled observations, we use “**x**” to indicate an observation (consisting of multiple attributes, such as surface velocity and surface temperature), “*s*” to indicate whether the observation is labeled (*s* = 1) or unlabeled (*s* = 0), and “*y*” to indicate the value of the label (*y* = 1 for a positive label and *y* = 0 for a negative label). Given the absence of negative observations for training, if the observation of the training set is labeled, the label will be positive for certain

p(y=1∣s=1)=1 (1)

Using KDE, the multivariate densities of the positive and the unlabeled observations are estimated, i.e., *p*(**x**|*s* = 1) and *p*(**x**|*s* = 0). These densities are evaluated on the observation that needs to be classified and are then transformed into the posterior probability that the observation is labeled or unlabeled, i.e., *p*(*s* = 1|**x**) and *p*(*s* = 0|**x**). To derive the probabilities, Bayes’ rule and the law of total probability are used

$$\begin{array}{c} p(s=1|x)=\frac{p(x|s=1)\times p(s=1)}{p(x)}=\\ \frac{p(x|s=1)\times p(s=1)}{p(x|s=1)\times p(s=1)+p(x|s=0)\times p(s=0)} \end{array}$$ (2.1)

and

p(s=0∣x)=1−p(s=1∣x) (2.2)

Our final objective is to determine the probability that an observation is positive or negative, i.e., *p*(*y* = 1|**x**) and *p*(*y* = 0|**x**). To obtain these values, the priors *p*(*s* = 1) and *p*(*s* = 0) are adjusted. These priors are obtained by calculating the proportion of labeled and unlabeled observations, respectively. The ratio of positive and negative observations exceeds the ratio of labeled and unlabeled observations, as not all positive observations are labeled. Therefore, the priors *p*(*s* = 1) and *p*(*s* = 0) are scaled using a cost parameter as in Bayes’ decision rule of minimum risk

$$\begin{array}{cc} p(y=1)=\frac{n_{\text{labeled}}\times \lambda }{n_{\text{labeled}}\times \lambda +n_{\text{unlabeled}}} & \text{and}\;p(y=0)=\frac{n_{\text{unlabeled}}}{n_{\text{labeled}}\times \lambda +n_{\text{unlabeled}}} \end{array}$$ (3)

where *n*_labeled_ is the number of labeled observations, *n*_unlabeled_ is the number of unlabeled observations, and λ is the cost parameter. The estimates for *p*(*y* = 1) and *p*(*y* = 0) are used in Eq. 2 instead of *p*(*s* = 1) and *p*(*s* = 0), respectively, to approximate *p*(*y* = 1|**x**) and *p*(*y* = 0|**x**)

$$p(y=1|x)\approx \frac{p(x|s=1)\times p(y=1)}{p(x|s=1)\times p(y=1)+p(x|s=0)\times p(y=0)}$$ (4.1)

and

$$p(y=0|x)\approx \frac{p(x|s=0)\times p(y=0)}{p(x|s=1)\times p(y=1)+p(x|s=0)\times p(y=0)}$$ (4.2)

Observations are classified as positive when

p(y=1∣x)>p(y=0∣x),which equals p(y=1∣x)>0.5 (5)

**Fig. 7.**
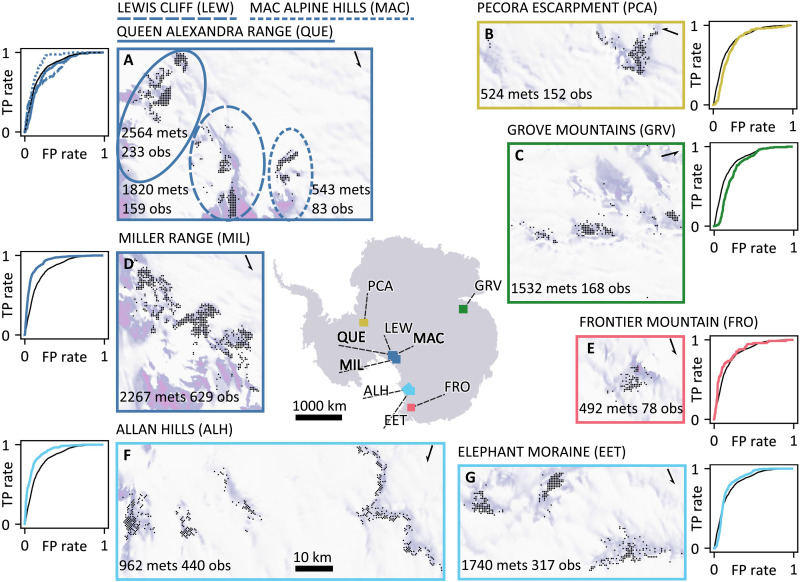
Positive observations for the nine most productive field sites and results of the cross-validation based on the four selected features (surface temperature, surface velocity, radar backscatter, and surface slope). The Antarctic map shows the location of the field sites. The submaps [(**A** to **G**), all on the same scale] display the positive observations as black dots, with the number of meteorite finds indicated with “mets,” and the resulting number of reprojected positive observations indicated with “obs.” The corresponding ROC curves (FP = false positive; TP = true positive), obtained with the actual negative observations (Fig. 3), indicate the performance of each individual field site (colored) compared to the weighted average ROC curve (black). The weighted average ROC curve is obtained by averaging the individual field sites, where the number of meteorite finds (mets) is used as a weight.

Before classifying observations, the classifier is calibrated to select predictive features (see the “Feature selection” section) using the ROC curve (Fig. 3C). This ROC curve is constructed with the calibration data and different values of λ through a cross-validation procedure. For 10-fold cross-validation, the positive observations (reprojected meteorite finding locations) are subdivided into 10 sets of observations, which represent the nine most productive field sites, and all remaining observations (Fig. 7). The classifier is trained successively with all but one of the 10 sets of positive observations. The unused positive observations, together with a set of negative observations, and arbitrary values for λ, are then used to estimate the true-positive and false-positive rates, which are combined in the ROC curve (Fig. 3C). Consequently, each point on the ROC curve (so-called “operating point”) corresponds to a value of λ. The 10-fold cross-validation procedure thus results in 10 different ROC curves from which an average curve is calculated (Fig. 7). To compare the performance of the classifier in different settings, the AUC is used, where a larger area means a better performance (Fig. 3C).

To finally classify observations as positive or negative, a single point on the ROC curve, corresponding to a value of the cost λ, needs to be selected. Selecting an operating point implies a trade-off between the false-positive and true-positive rates. On the one hand, for low false-positive rates, the precision of the classifier is high, but the sensitivity is low. On the other hand, for high true-positive rates, the sensitivity of the classifier is high, but the precision is low. The trade-off between sensitivity and precision is quantified by maximizing the harmonic mean of the two metrics (i.e., maximizing the F1 score). Here, the precision and sensitivity are estimated using the true-positive and false-positive rates obtained during the cross-validation (using the actual negative data, dark blue in Fig. 3 and table S1). In summary, the optimal operating point is obtained empirically by varying the cost λ that reweighs the positive class prior to accommodate for the positive and unlabeled data. The optimal value for λ was found to equal 200.

The classifying algorithm, written in Python using the Scikit-learn library, consists of three steps. (i) First, the input data are transformed (see the “Data for features” section) and standardized for the mean to be 0 and the variance to be equal to 1. Then, a principal component analysis is performed to reduce the dimensionality of the data. The dimensionality of the data needs to be reduced, as the number of investigated features equals six, while to obtain a reliable estimate of the density using the limited number of positive observations (2,554), the dimension of the data should be reduced to five (*56*). (ii) The multidimensional densities of the first five (or less, depending on the dimensionality of the observations) principal components of the labeled and unlabeled observations are estimated through a KDE with a 10-fold cross-validation to obtain the bandwidth parameter. For the KDE of the unlabeled observations, a random selection of 10,000 observations is used. (iii) The decision rule (Eqs. 3 to 5) is applied to classify observations as positive or negative.

### Post-processing

To obtain the outlines of the classified MSZs, the classified pixels are post-processed using the GIS-software QGIS and GRASS. First, isolated positive and negative pixels are eliminated arbitrarily by applying a majority filter three times. The first two filtering steps use a circular kernel with a two-pixel radius, while the third step consists of using a one-pixel radius circular kernel. Subsequently, smooth outlines of the filtered results are obtained by vectorizing the raster data and smoothing the edges iteratively using a Chaiken filter with a 350-m tolerance value (10 iterations).

### Where-to-go index

The where-to-go index, used to rank MSZs, is calculated by considering three parameters that are important for a successful field mission. For each of the three parameters, the rank of the MSZ is calculated and subsequently summed up to obtain the where-to-go index. Hence, MSZs with low where-to-go indices correspond to MSZs with a high potential, and vice versa.

The three considered parameters are as follows: (i) the distance to the nearest research station, which is computed in QGIS using a dataset of Antarctic Facilities (COMNAP 2019, v3.3.0, released 4 May 2020), where only “open” stations are considered. In defining this parameter, we prioritized simplicity and interpretability, as the true logistic complexity of a field visit is impossible to summarize in a single quantitative value. (ii) The median of the a posteriori probability of all grid cells within the (potential) MSZ. The a posteriori probability relates to the probability to find meteorites at a given location (Fig. 5; see the “Classifier” section). (iii) A parameter representing the presence of temporary snow layers. This parameter corresponds to the number of days during the meteorite collecting season (November to March) for which at least 50% of the MSZ is snow free. For large MSZs (>20 km^2^), we use an alternative criterion: here, at least 10 km^2^ should be snow free. To estimate whether a location (within an MSZ) is snow free, we use the shortwave white sky albedo from the MCD43A3 MODIS Albedo Daily 500-m product (*57*). A threshold between snow and ice was found by performing various tests and was set at 0.72; values smaller than this threshold are considered to represent snow-free days. No measurements have been disregarded using the quality bands, because the uncertainties are assumed to be reduced by considering a large time span (18 February 2000 until 9 February 2021) and a uniform number of measurements over the entire continent is preferred. Despite potential biases introduced by considering all data (*58*, *59*), this approach is considered to be sufficient for the specific purpose.

## Supplementary Materials

### This PDF file includes:

Figs. S1 to S7 Tables S1 to S5 Legend for table S6 References
