# Supplementary material for: Unexplored Antarctic meteorite collection sites revealed through machine learning
Source: Sci Adv. 2022 Jan 26;8(4):eabj8138. doi: 10.1126/sciadv.abj8138 (PMC8791461; doi:10.1126/sciadv.abj8138)
Supplement: Supplementary file 1 — Figs. S1 to S7 Tables S1 to S5 Legend for table S6 References [file sciadv.abj8138_sm.pdf]

Science Advances
AAAS

Supplementary Materials for

Unexplored Antarctic meteorite collection sites revealed through machine learning

Veronica Tollenaar*, Harry Zekollari, Stef Lhermitte, David M.J. Tax, Vinciane Debaille, Steven Goderis, Philippe Claeys, Frank Pattyn

*Corresponding author. Email: veronica.tollenaar@ulb.be

Published 26 January 2022, *Sci. Adv.* **8**, eabj8138 (2022)
DOI: 10.1126/sciadv.abj8138

The PDF file includes:

Figs. S1 to S7
Tables S1 to S5
Legend for table S6
References

Other Supplementary Material for this manuscript includes the following:

Table S6

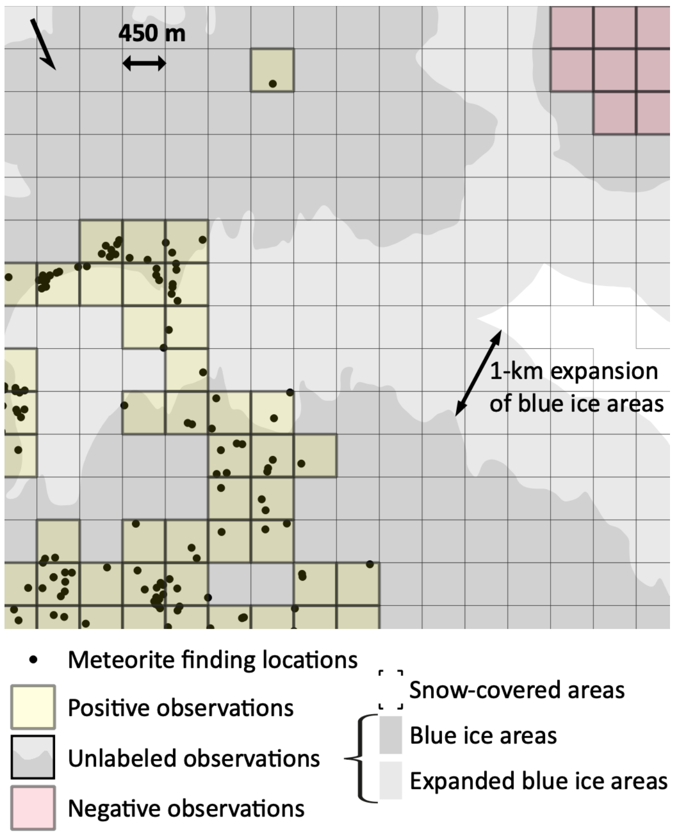

Fig. S1. Definition of observations, illustrated for an area near Elephant Moraine ($76^0 17$'S, $157^0 20$'E). When one or multiple meteorites are found within a grid cell, the observation is labelled positive. Unlabeled observations are the centers of regularly spaced grid cells overlaying blue ice areas (BIAs). The negative observations are extracted from the set of unlabeled observations, using information retrieved from fieldwork reports.

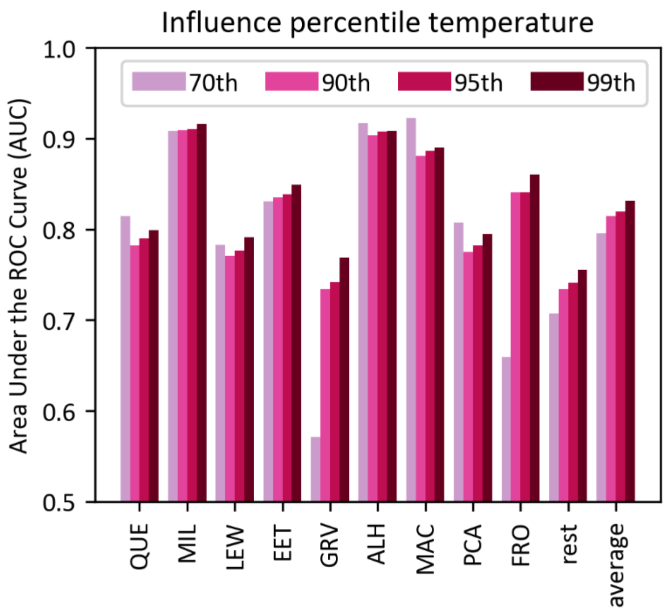

Fig. S2. Influence of the definition of the surface temperature feature on the classification. The area under the ROC curve (AUC) is calculated using the classification based on the four selected features (surface temperature, surface velocity, radar backscatter, and surface slope over 2.2 km) and negative validation data. The variation of the definition of the surface temperature resides in the selected percentile of 19 years of 8-daily observations. The 99th percentile performs best (largest AUC), indicating that very rare extreme heat events are relevant in predicting the presence or absence of meteorites at a given location.

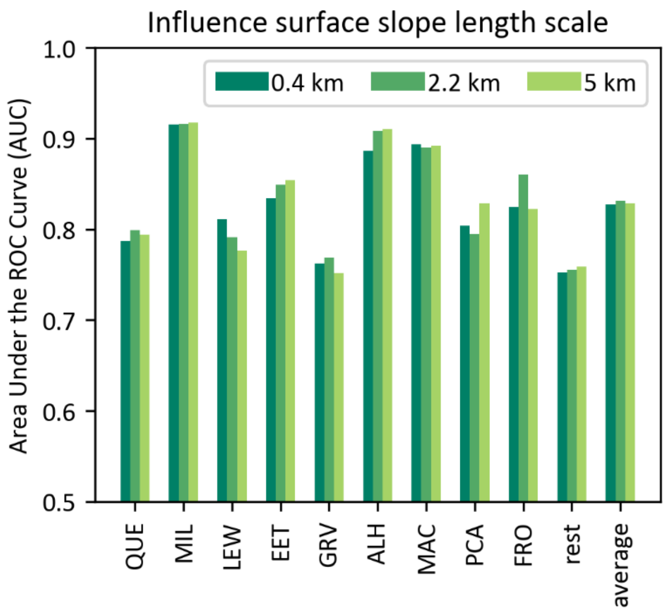

Fig. S3. Influence of the definition of the surface slope feature on the classification. The AUC is calculated using the classification based on the four selected features (surface temperature (99th percentile), surface velocity, radar backscatter, and surface slope) and negative validation data. The variation of the definition of the surface slope resides in the selected diameter of the filtering footprint (see "Data for features" in "Methods"), i.e., the distance (length scale) over which the surface slope is calculated. The average performance of the classifier is relatively unsensitive to the different definitions of the surface slope.

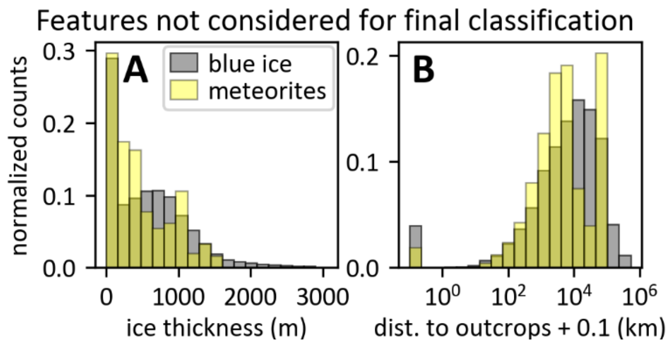

Fig. S4. Histograms of the two features that were not selected during the feature selection procedure: (**A**) ice thickness (*36*, *39*) and (**B**) distance to outcrops (*40*) (see "Data for features" in "Methods"). Values for the ca. 2.1 million unlabeled observations at the expanded blue ice areas are in grey ("blue ice") and the 2,554 positive observations are in yellow ("meteorites").

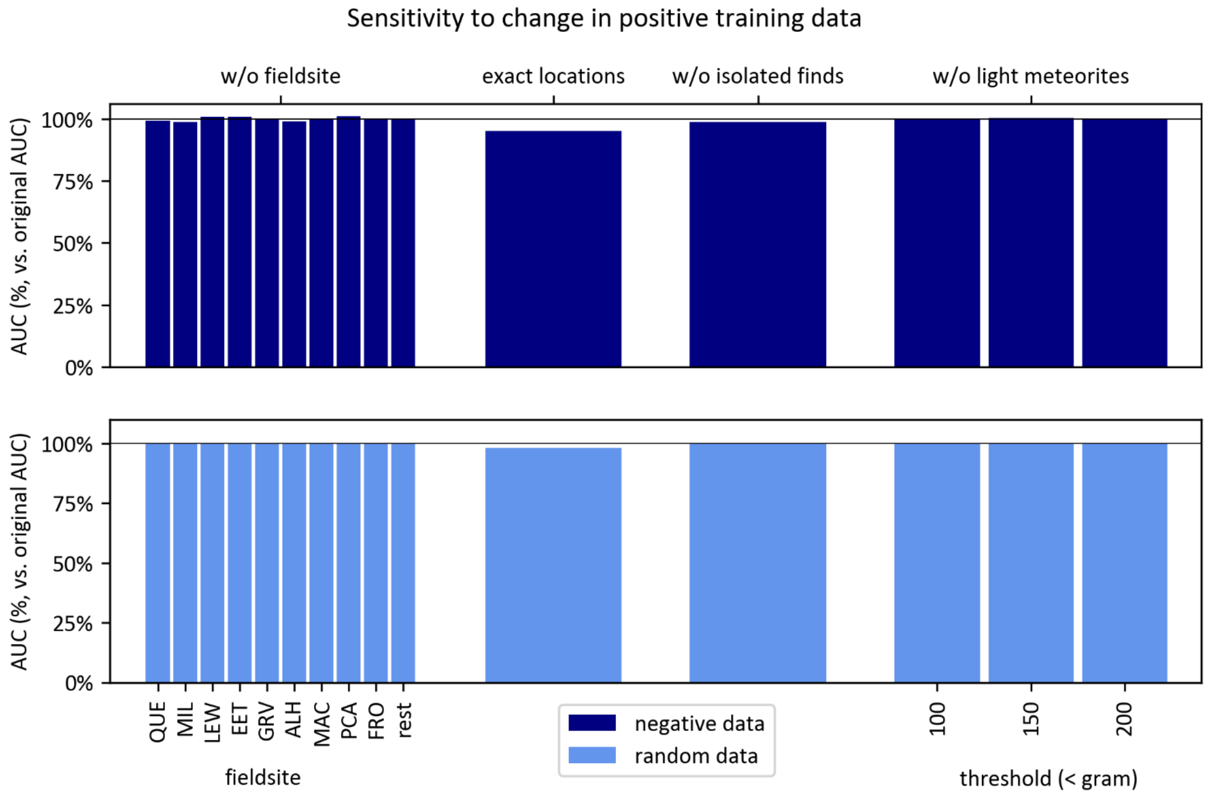

Fig. S5. Change in the AUC resulting from a change in the positive training data. On the y-axes the relative AUC of the alternated classification compared to the AUC of the original classification. In the upper panel, the calibration data to obtain the AUCs consist of positive observations and negative observations, while for the lower panel, positive observations and randomly selected unlabeled observations are used (see “Feature selection” in “Results”). Four sets of differently defined positive observations are investigated (upper x-axis). (i) W/o fieldsite, i.e., all observations of any of the nine most productive field sites are disregarded (respective field sites are indicated on the lower x-axis). (ii) Exact locations, i.e., the exact finding locations of the meteorites are used. (iii) W/o isolated finds, i.e., grid cells containing only a single meteorite find are disregarded. (iv) W/o light meteorites, i.e., meteorites lighter than a certain threshold (indicated on the lower x-axis) are disregarded.

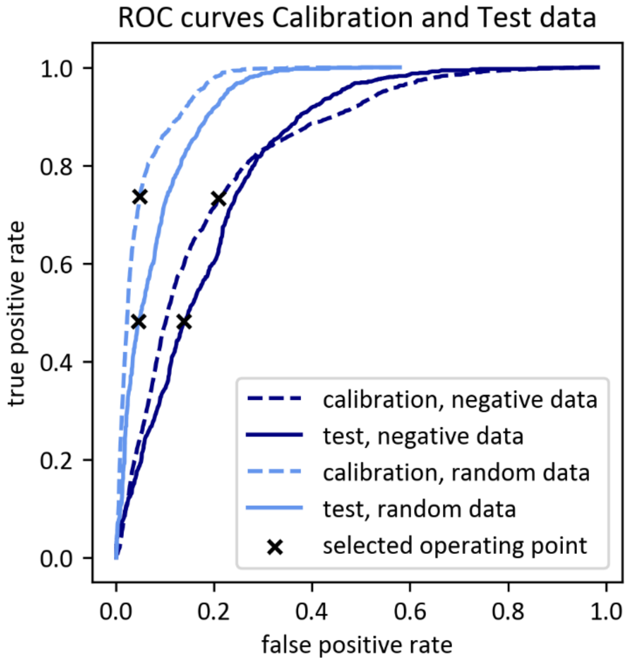

Fig. S6. ROC curves obtained with calibration data and with test data. Two sets of calibration data are used, one with actual negative observations (dark blue) and one with randomly selected unlabeled observations (light blue; see "Feature selection" in "Methods"). To obtain comparable ROC curves in the (grid-cell level) evaluation, two sets of independent test data are used (indicated with negative data, dark blue, and random data, light blue). The selected operating point is displayed on all curves. As this point corresponds to a fixed value of the cost parameter λ, the location of the operating points is on different places of the ROC curves. The ROC curves of the test data are slightly outperformed by the training ROC curves (the former have a lower AUC), indicating that the classifier is not overfitted. The corresponding reduction of the AUC is 2% for the negative data and 3% for the random data.

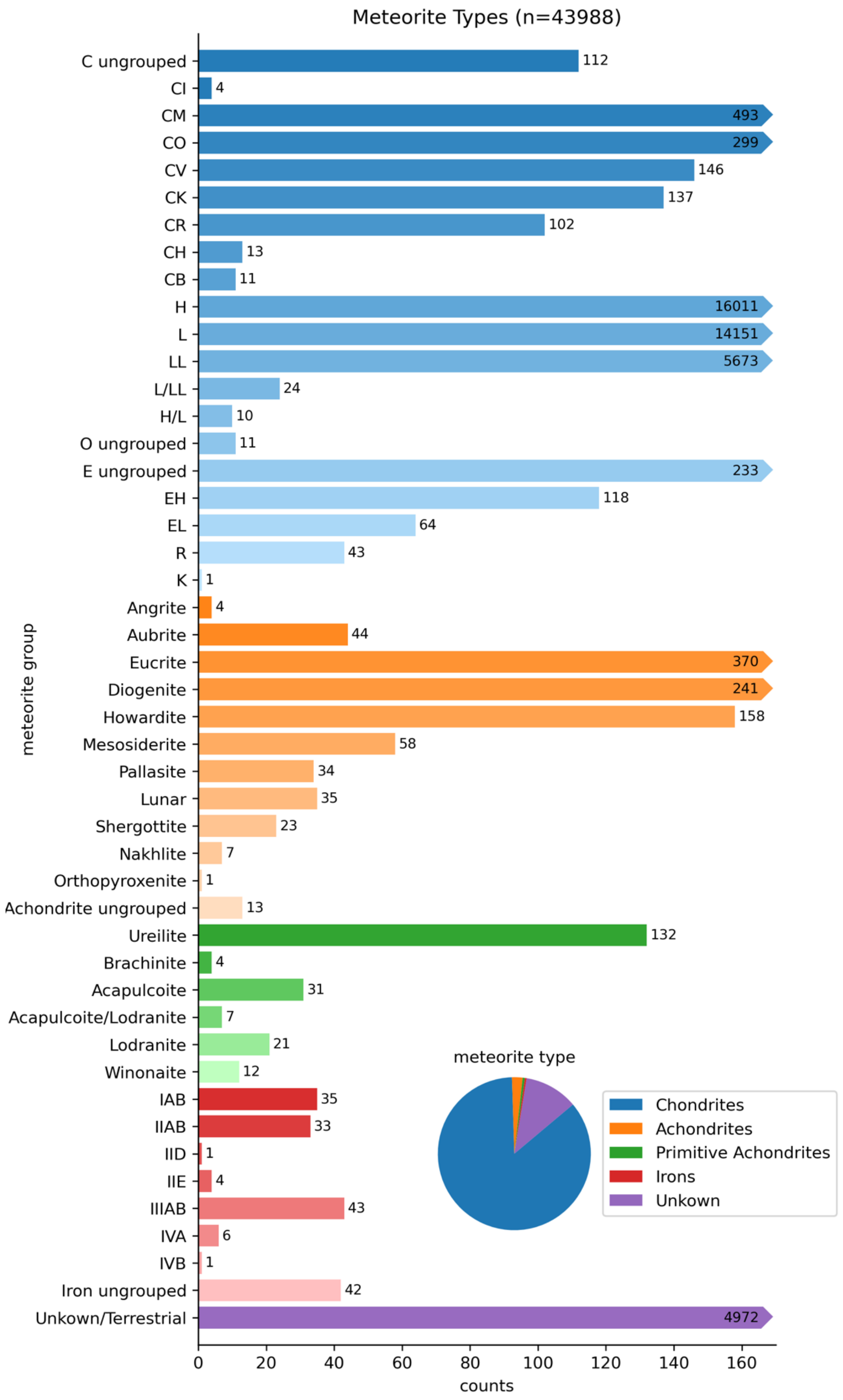

Fig. S7. Antarctic meteorite types and groups as provided in the Meteoritical Bulletin Database (*6*), as consulted on 05/07/2019. Note that counts exceeding 160 are indicated with arrows. The colors of the meteorite groups (bars) correspond to the colors of the meteorite types (pie diagram).

**Metric**	Equation	Calibration data with negative observations	Calibration data with random observations
Precision	$\frac{TP}{TP + FP}$	50.7 %	81.1 %
Sensitivity	$\frac{TP}{TP + FN}$	73.3 %	73.7 %
Specificity	$\frac{TN}{TN + FP}$	79.2 %	93.2 %
Accuracy	$\frac{TP + TN}{TP + TN + FP + FN}$	77.8 %	90.4 %

Table S1. Performance metrics of the calibration. In the column "Equation", TP denotes the number of true positives, FP the number of false positives, FN the number of false negatives, and TN the number of true negatives. The column "Calibration data with negative observations" gives the estimated metrics using calibration data consisting of positive observations and actual negative observations deduced form fieldwork reports. With this set of calibration data, the operating point of the final classification has been selected by maximizing the harmonic mean of the precision (F1 score). The column "Calibration data with random observations" gives the estimated metrics using calibration data consisting of positive observations and randomly selected unlabeled observations, reflecting the capability of the classifier to distinguish positive observations from arbitrary areas within the expanded blue ice areas (such as exposed rocks or snow-covered areas).

	Observed positive	Observed negative	Total
**Classified positive**	469 (TP)	522 (FP)	991
**Classified negative**	504 (FN)	3248 (TN)	3752
**Total**	973	3770	

Table S2. Confusion matrix at the grid-cell level. The confusion matrix is obtained by using independent test data consisting of positive and negative observations on the same 450-meter grid as the training data.

MSZ (abbreviation of field site)	Location	Number of meteorite finds	Amount of positive training obs.	Surface temperature (99th percentile)	Surface velocity	Radar backscatter	Surface slope (over 2.2 km)	Classified (yes=1, part.=0.5, no=0)
Belgica (B)	72.546°S, 31.249°E	37	0	-12.3 °C	2.7 m/yr	160	57.8 m/km	0
Buckley Island (BUC)	84.984°S, 163.818°E	29	0	-12.5 °C	3.2 m/yr	178	53.8 m/km	0.5
Cumulus Hills (CUM)	85.478°S, -174.998°E	79	0	-14.8 °C	3.7 m/yr	162	34.3 m/km	1
Derrick Peak (DRP)	80.092°S, 156.464°E	27	0	-6.8 °C	7.8 m/yr	172	39.6 m/km	0
Dominion Range (DOM)	85.339°S, 164.524°E	2097	0	-14.7 °C	17.1 m/yr	169	24.7 m/km	1
Geologists Range (GEO)	82.698°S, 155.289°E	32	0	-11.9 °C	13.0 m/yr	171	34.2 m/km	0.5
La Paz Icefield (LAP)	86.329°S, -70.385°E	1654	0	-15.0 °C	4.1 m/yr	134	9.8 m/km	1
Larkman Nunatak (LAR)	85.711°S, 179.192°E	1020	0	-18.8 °C	1.5 m/yr	162	15.5 m/km	1
Meteorite City (Elephant Moraine, EET)	76.22°S, 156.632°E	438	5	-14.7 °C	2.6 m/yr	130	10.2 m/km	1
Meteorite Hills (MET)	79.636°S, 155.331°E	1129	0	-14.7 °C	6.0 m/yr	163	23.0 m/km	0.5
Mount Pratt (PRA)	85.412°S, 176.6°E	22	0	-19.7 °C	1.5 m/yr	154	35.9 m/km	1
Mt Ward Ice Tongue (Dominion Range, DOM/Grosvenor Mountains, GRO)	85.65°S, 166.906°E	27	0	-16.5 °C	1.0 m/yr	159	42.8 m/km	1
Nansen A (Asuka, A)	72.741°S, 23.981°E	794	0	-17.1 °C	1.2 m/yr	149	18.6 m/km	1
Nansen B (Asuka, A)	72.899°S, 24.256°E	1066	0	-19.2 °C	1.4 m/yr	147	13.2 m/km	1
Nansen C (Asuka, A)	72.79°S, 24.874°E	433	0	-20.2 °C	1.3 m/yr	123	14.5 m/km	1
North Forty (Pecora Escarpment, PCA)	85.348°S, -70.288°E	62	3	-12.5 °C	2.2 m/yr	96	7.9 m/km	1
Roberts Massif (RBT)	85.609°S, -177.106°E	229	0	-15.0 °C	4.0 m/yr	172	37.6 m/km	1
Scott Glacier (SCO)	87.052°S, -147.766°E	42	0	-16.9 °C	1.3 m/yr	149	13.5 m/km	1
Szabo Bluff (SZA)	86.469°S, -145.012°E	44	0	-13.9 °C	2.1 m/yr	132	33.3 m/km	1
Total: **19 MSZs**		9261	8					15.5

Continued on next page

Non-MSZ	Location	Number of unlabeled observations	Percentage of area classified as MSZ	Surface temperature (99th percentile)	Surface velocity	Radar backscatter	Surface slope (over 2.2 km)	Classified (yes=1, part.=0.5, no=0)
A140-A180	72.468°S, 23.249°E	1071	26%	-14.3 °C	7.1 m/yr	166	19.1 m/km	1
above Morris Cliff	80.314°S, -81.906°E	11	0%	-10.8 °C	6.4 m/yr	140	98.8 m/km	0
Between Mt. Simmons and Mt. Geissel	80.391°S, -81.637°E	4	0%	-12.6 °C	9.6 m/yr	155	40.5 m/km	0
Independence Hills	80.382°S, -81.509°E	111	0%	-8.3 °C	8.2 m/yr	111	28.3 m/km	0
Marble Hills	80.247°S, -82.079°E	47	0%	-8.3 °C	6.2 m/yr	117	18.0 m/km	0
Martin Hills	82.053°S, -87.947°E	14	0%	-14.1 °C	4.4 m/yr	114	31.4 m/km	0
Minaret Bowl	80.246°S, -82.297°E	16	0%	-11.8 °C	4.6 m/yr	110	28.4 m/km	0
Morris Cliff	80.293°S, -81.833°E	75	0%	-7.7 °C	5.7 m/yr	115	17.3 m/km	0
Mount Bamse	72.422°S, 21.952°E	1118	0%	-10.9 °C	16.1 m/yr	183	25.9 m/km	0
Mount Nils Larsen	72.361°S, 22.674°E	535	5%	-10.9 °C	7.0 m/yr	163	31.3 m/km	0
Pattriot Hills	80.318°S, -81.379°E	59	0%	-7.3 °C	7.2 m/yr	120	18.9 m/km	0
Pirrit Hills	81.146°S, -85.227°E	63	0%	-10.5 °C	7.2 m/yr	105	22.4 m/km	0
Sequence Hills	73.038°S, 161.284°E	80	55%	-10.7 °C	1.6 m/yr	116	23.1 m/km	1
Total: **13 non-MSZs**		3204	-					2

Table S3a. MSZs and non-MSZs used in the evaluation at the MSZ level. The location of the MSZs is obtained by using the List of Dense Meteorite Collection Areas published by the Meteoritical Society (*6*), occasional coordinates of meteorite finds (*6*) and maps of Antarctic Meteorite Recovery Locations published by NASA (*64*). The references of the location of the non-MSZs are listed in Table S5. The negligible amount of positive observations (i.e., single observations on the 450-meter grid) used for training is listed for completeness. For the non-MSZs, the amount of unlabeled gridded observations is listed. The mean values of the features within the MSZ/non-MSZ (surface temperature, surface velocity, radar backscatter and surface slope) are indicated, but as the exact outlines of the MSZs/non-MSZs are often not known exactly, these estimates might be slightly biased. The last column indicates whether an observation is (partially) classified or not.

	Observed MSZ	Observed non-MSZ	Total
**Classified MSZ**	15.5 (TP)	2 (FP)	17.5
**Classified non-MSZ**	3.5 (FN)	11 (TN)	14.5
**Total**	19	13	

Table S3b. Confusion matrix at the MSZ level. The confusion matrix is obtained by using independent test data consisting of MSZs and non-MSZs as listed in Table S3a.

MSZ/area	Source + remarks
Meteorite Hills	Literature (*65*) (reprojected map)
Mount Balchen	Literature (*66*) (reprojected map)
Nansen (Asuka)	Literature (*67*) (reprojected map)
Yamato	Literature (*4*) (reprojected map)
Antarctica (no field site indicated; 4 finds)	Meteoritical Bulletin Database (*6*)
Asuka (5 finds)	
Devils Glacier (2 finds)	
Dominion range (31 finds)	
Elephant Moraine (167 finds)	
Grosvenor Mountains (173 finds)	
ßLaPaz Icefield (1 find)	
Mount Prestrud (4 finds)	
Nodtvedt Nunatak (39 finds)	
Yamato (557 finds)	

Table S4. Positive observations used for testing. These positive observations are partly obtained by georeferencing maps provided in the literature and manually labelling the positive grid cells (source: Literature). The remaining positive observations consist of meteorite finding locations published in the Meteoritical Bulletin Database (*6*) after 05/07/2019 (source: Meteoritical Bulletin Database). The number of meteorite finds published in the database is indicated. However, the number of positive observations used for testing is reduced by reprojecting the exact finding locations and eliminating positive observations that overlap with the training data (to guarantee the independence of the testing data).

Name	Location	Information on search
**CALIBRATION DATA**		
Allan Hills Northwestern icefield	76.72°S, 156.058°E	Small and separate blue ice area approximately 16 kilometers north of the far western icefield in Allan Hills. "Thorough reconnaissance search" in 1985-86 ANSMET season, only one meteorite find (*4*, *68*)
Battlements Nunatak icefields (2 parts)	76.511°S, 159.398°E	Large, exposed icefields around Battlements Nunatak. Reconnaissance search in 1984-85 ANSMET season (*69*)
Bessinger Nunatak icefield	85.09°S, -64.729°E	Extensive searching in 1991-92 ANSMET season, only two meteorites found (*70*)
Boomerang Ranges icefield	77.578°S, 160.332°E	Careful search by helicopter in the 1978-79 ANSMET season (*4*, *71*)
Brimstone Peak icefield	78.526°S, 158.879°E	Blue ice located downstream of the outcrop Brimstone Peak. Visited by snowmobiles in the 1980-81 ANSMET season (*72–74*)
Butcher Ridge icefield	75.787°S, 158.592°E	Careful search by helicopter in the 1978-79 ANSMET season (*71*, *75*)
Carapace Nunatak icefield	79.169°S, 155.969°E	Helicopter-assisted survey of some smaller patches near Carapce Nunatak in 1976-77 season, visited in 1981-82 ANSMET season, many geodes at base of Carapace Nunatak, no meteorites found (*63*, *76*)
Colbert Hills icefield	76.877°S, 159.462°E	Above and below submerged parts of Colbert Hills. Only one meteorite find below ice ramp, supports view that main body Walcott Névé is not a productive area (*4*)
David Glacier icefield	84.194°S, 162.604°E	Along north facing escarpments there are areas of blue ice, not clear if all BIAs around David Glacier are meant. Five days of rigorous searching in ANSMET season 1992-93 (*77*)
Emlen Peaks icefields (6 parts)	75.693°S, 155.284°E	Bare ice patches at Emlen Peaks, downstream. Reconnaisance by helicopter with occasional ground checks in 1981-82 ANSMET season (*78*)
Far Northern ice patches (2 parts)	71.888°S, 160.583°E	Side by side traverse on snowmobiles in 1987-88 ANSMET season (*79*)
Finger Ridge icefield	75.863°S, 156.23°E	Careful search by helicopter in the 1978-79 ANSMET season (*71*, *75*)
Gallipoli Heights ice patches (2 parts)	79.128°S, 156.806°E	Snowmobile traverse in 1981-82 ANSMET season (*78*)
Gordon Valley icefield	72.433°S, 163.725°E	Some blue icefields along edge of Queen Alexandra Range in Walcott Névé. Only two specimens encountered, supports view that main body Walcott Névé is not a productive area (*4*)
Griffin Nunatak icefield	84.38°S, 163.835°E	Blue ice located downstream of the outcrop Griffin Nunatak(*72*), icefield near Ambalada Peak (*73*). Visited by snowmobiles in the 1980-81 ANSMET season (*72–74*)
Ice patch between Mount Baldr and Mount Fleming	75.912°S, 158.548°E	Wright Upper Glacier receives small amount of ice from area between Mount Baldr and Mount Fleming. Two isolated finds when flying over by helicopter, searched this area and Wright Upper Glacier for six weeks during 1976-77 ANSMET season (*4*, *63*, *80*)
Jarina Nunatak to Trinity Nunatak icefields (4 parts)	76.41°S, 160.36°E	Searched in 1985-86 ANSMET season with no success (*68*)
Largest icefield along Davies Escarpment	85.499°S, -89.786°E	Extensive search in 1982-83 ANSMET season, six meteorite specimens found in northern part of icefield, “search of this icefield indicates that large concentrations of meteorites do not exist on it” (*81*, *82*)
Lekander Nunatak icefield	85.036°S, -64.793°E	Few isolated meteorite finds (*4*)

Continued on next page

Name	Location	Information on search
Lewis Nunatak icefields (3 parts)	85.734°S, -88.447°E	Searched by snowmobiles in 1982-83 ANSMET season, only one meteorite found below ice cliffs (*81*, *82*)
Lonely One Nunatak icefield	71.185°S, 161.316°E	Bare ice patch at Lonely One Nunatak, downstream. Reconnaissance by helicopter with occasional ground checks in 1981-82 ANSMET season (*78*)
Lonewolf Nunataks icefield	81.334°S, 152.633°E	Careful search by helicopter in the 1978-79 ANSMET season (*71*, *75*)
Manhaul Bay icefield	76.643°S, 159.725°E	Ice patch between arms of Allan Hills. Two isolated meteorite finds, one in 1977-78 ANSMET season, when foot searches were conducted (*4*, *83*)
Monument Nunataks icefields (6 parts)	72.563°S, 162.265°E	Bare ice patches at Monument Nunataks, downstream. Reconnaisance by helicopter with occasional ground checks in 1981-82 ANSMET season (*78*)
Mount Crean icefield	77.849°S, 159.682°E	Helicopter-assisted survey in 1676-77 ANSMET season (*63*)
Mount Dewitt icefield	77.199°S, 159.811°E	Extensive plateau area of blue ice around Mount Dewitt. Searched in 1676-77 ANSMET season (*63*)
Mount Howe icefield	87.317°S, -149.956°E	Icefield of ca. 18 km^2 at west-facing foot of Mount Howe. Extensive week-long search in 1988-89 ANSMET season by two team members, only four specimens found (*4*, *84*)
Mount Tolchin icefield	85.09°S, -65.287°E	Few isolated meteorite finds (*4*)
Onlooker Nunatak icefield	71.909°S, 162.371°E	Reconnaissance by helicopter with occasional ground checks in 1981-82 ANSMET season (*78*)
Outback Nunataks icefields (16 parts)	72.596°S, 160.64°E	Bare ice patches at Outback Nunataks, downstream. Reconnaisance by helicopter with occasional ground checks in 1981-82 ANSMET season (*78*)
Outpost Nunatak icefield	75.816°S, 158.21°E	Blue ice located downstream of the outcrop Outpost Nunatak. Visited by snowmobiles in the 1980-81 ANSMET season, found one isolated meteorite specimen (*4*, *72*, *74*, *76*)
Patuxent Main icefield	84.653°S, -62.344°E	Expansive blue ice area (ca. 300 km^2) east of Anderson Hills(*70*)/Patuxent Mountains (*4*, *49*). Five days exploring in 1991-92 ANSMET season, only 22 meteorites recovered (*4*, *49*, *70*)
Renerie Rocks icefield	71.303°S, 161.365°E	Bare ice patch at Renerie Rocks, downstream. Reconnaissance by helicopter with occasional ground checks in 1981-82 ANSMET season (*78*)
Scharffenbergbotnen (2 parts)	74.571°S, -11.128°E	Heimefrontfjella. No details on search indicated (*85*)
Shooda Bin icefield	76.332°S, 156.897°E	Bowl-shaped large icefield. Reconnaissance trip in 1996-97 ANSMET season, only five specimens in large area (*86*)
South and west of Reckling Peak	76.279°S, 159.3°E	Extensive areas south and west of the peak. Traversed in 1985-86 ANSMET season (*68*)
Tent Rock icefield	75.691°S, 158.568°E	Blue ice located downstream of the outcrop Tent Rock. Visited by snowmobiles in the 1980-81 ANSMET season (*72*, *74*, *76*)
Turnstile Ridges icefield	79.811°S, 154.735°E	Careful search by helicopter in the 1978-79 ANSMET season (*71*, *75*)
Unnamed Nunatak icefield	84.861°S, -69.124°E	Small local blue ice patches at an unnamed nunatak (84°51'S 68°40'W). Few hours exploring in 1991-92 ANSMET season (*70*)
Upstream icefield of Chastain Peak	85.187°S, -94.816°E	Searched by snowmobiles in 1982-83 ANSMET season (*81*, *82*)
Westhaven Nunatak icefield	79.855°S, 154.308°E	Careful search by helicopter in the 1978-79 ANSMET season (*71*, *75*)
Wright Upper Glacier	77.54°S, 160.649°E	Debris at the bottom end of Wright Upper Glacier. Searched some days in the terminal moraine (also with a mine detector), and also at the surface of the glacier in 1976-77 ANSMET season (*4*, *63*, *80*)

Continued on next page

Name	Location	Information on search
**TEST DATA**		
A 140-A 180	72.468°S, 23.249°E	Large area of bare ice south of A 140-A 180, 50 km long E-W and 5 km wide. Searched by six members of the Asuka party in mid-October, as fourth exploration of the 29th Japanese Antarctic Research Expedition (JARE-29), 1987-89 (*45*)
above Morris Cliff	80.314°S, -81.906°E	Assessed by aircraft and searched on foot in the 1997-1998 field in the context of the Robotic Antarctic Meteorite Search (RAMS) Program (*44*)
Between Mt. Simmons and Mt. Geissel	80.391°S, -81.637°E	Assessed by aircraft and searched on foot in the 1997-1998 field in the context of the Robotic Antarctic Meteorite Search (RAMS) Program (*44*)
Independence Hills	80.382°S, -81.509°E	Traversed and searched in the 1997-1998 field season by snowmobile and on foot in the context of the Robotic Antarctic Meteorite Search (RAMS) Program (*44*)
Marble Hills	80.247°S, -82.079°E	Traversed and searched in the 1997-1998 field season by snowmobile and on foot in the context of the Robotic Antarctic Meteorite Search (RAMS) Program (*44*)
Martin Hills	82.053°S, -87.947°E	Foot search in November 1998 in the context of the Robotic Antarctic Meteorite Search (RAMS) Program, searched in January 2007 in the context of the first Korea Expedition for Antarctic meteorites (KOREAMET) (*42*, *43*)
Minaret Bowl	80.246°S, -82.297°E	Traversed and searched in the 1997-1998 field season by snowmobile and on foot in the context of the Robotic Antarctic Meteorite Search (RAMS) Program (*44*)
Morris Cliff	80.293°S, -81.833°E	Traversed and searched in the 1997-1998 field season by snowmobile and on foot in the context of the Robotic Antarctic Meteorite Search (RAMS) Program (*44*)
Mount Bamse	72.422°S, 21.952°E	Bare icefield south of Mt. Bamse and Mt. Nils Larsen. Searched by six members of the Asuka party in mid-October, as fourth exploration of the 29th Japanese Antarctic Research Expedition (JARE-29), 1987-89 (*45*)
Mount Nils Larsen	72.361°S, 22.674°E	Searched by six members of the Asuka party in mid-October, as fourth exploration of the 29th Japanese Antarctic Research Expedition (JARE-29), 1987-89 (*45*)
Patriot Hills	80.318°S, -81.379°E	Traversed and searched in the 1997-1998 field season by snowmobile and on foot and visited again in November 1998 in the context of the Robotic Antarctic Meteorite Search (RAMS) Program (*42*, *44*)
Pirrit Hills	81.146°S, -85.227°E	Foot search in November 1998 in the context of the Robotic Antarctic Meteorite Search (RAMS) Program, searched in January 2007 in the context of the first Korea Expedition for Antarctic meteorites (KOREAMET) (*42*, *43*)
Sequence Hills	73.038°S, 161.284°E	Similar to Frontier Mountain ice field, valleys open to the NE. Searched during EUROMET 1990/91 season, valley floors drowned in large meltwater lakes. (*87*)

Table S5. Negative observations. Contains negative observations used for the calibration of the classifier, and negative observations used for testing. Details on the search and the relevant references are indicated in the last column.

Table S6. (separate file) Classified MSZs. Ranked according to the value of the where-to-go index (see "Methods"). The maximum temperature and the ice flow velocity represent the mean value of the observations within the MSZ (see "Methods"). The snow-free days represents the number of days per field season (November-February) that at least 50% of the MSZ (or for MSZs larger than 20 km^2 at least 10 km^2) is snow free (see "Methods").

REFERENCES AND NOTES

1. M. Gounelle, M. Gounelle, Meteorites: International law and regulations. *Meteorit. Planet. Sci.* **54**, 2887–2901 (2019).

2. R. Bintanja, On the glaciological, meteorological, and climatological significance of Antarctic blue ice areas. *Rev. Geophys.* **37**, 337–359 (1999).

3. F. Hui, T. Ci, X. Cheng, T. A. Scambos, Y. Liu, Y. Zhang, Z. Chi, H. Huang, X. Wang, F. Wang, C. Zhao, Z. Jin, K. Wang, Mapping blue-ice areas in Antarctica using ETM+ and MODIS data. *Ann. Glaciol.* **55**, 129–137 (2014).

4. W. Cassidy, R. Harvey, J. Schutt, G. Delisle, K. Yanai, The meteorite collection sites of Antarctica. *Meteoritics* **27**, 490–525 (1992).

5. A. Sinisalo, J. C. Moore, Antarctic blue ice areas—Towards extracting palaeoclimate information. *Antarct. Sci.* **22**, 99–115 (2010).

6. Meteoritical Bulletin Database, Bulletin of classified and named meteorite samples (2021); www.lpi.usra.edu/meteor/ [accessed 2 February 2021].

7. M. Yoshida, H. Ando, K. Omoto, R. Naruse, Y. Ageta, Discovery of meteorites near Yamato Mountains, East Antarctica. *Antarct. Rec.* **39**, 62–65 (1971).

8. M. Yoshida, Discovery of the Yamato meteorites in 1969. *Polar Sci.* **3**, 272–284 (2010).

9. H. Zekollari, S. Goderis, V. Debaille, M. van Ginneken, J. Gattacceca, A. J. Timothy Jull, J. T. M. Lenaerts, A. Yamaguchi, P. Huybrechts, P. Claeys, Unravelling the high-altitude Nansen blue ice field meteorite trap (East Antarctica) and implications for regional palaeo-conditions. *Geochim. Cosmochim. Acta* **248**, 289–310 (2019).

10. L. Folco, A. Capra, M. Chiappini, M. Frezzotti, M. Mellini, I. E. Tabacco, The Frontier Mountain meteorite trap (Antarctica). *Meteorit. Planet. Sci.* **37**, 209–228 (2002).

11. G. Corti, A. Zeoli, M. Bonini, Ice-flow dynamics and meteorite collection in Antarctica. *Earth Planet. Sci. Lett.* **215**, 371–378 (2003).

12. R. Harvey, The origin and significance of Antarctic meteorites. *Chem. Erde* **63**, 93–147 (2003).

13. G. Corti, A. Zeoli, P. Belmaggio, L. Folco, Physical modeling of the influence of bedrock topography and ablation on ice flow and meteorite concentration in Antarctica. *J. Geophys. Res.* **113**, F01018 (2008).

14. G. W. Evatt, A. R. D. Smedley, K. H. Joy, L. Hunter, W. H. Tey, I. D. Abrahams, L. Gerrish, The spatial flux of Earth's meteorite falls found via Antarctic data. *Geology* **48**, 683–687 (2020).

15. R. P. Harvey, J. Schutt, J. Karner, Fieldwork Methods of the U.S. antarctic search for meteorites program, in *35 Seasons of the U.S. Antarctic Meteorites (1976–2010): A Pictorial Guide to the Collection*, K. Righter, C. M. Corrigan, T. J. McCoy, R. P. Harvey, Eds. (AGU and Wiley, 2014), pp. 23–41.

16. J. T. M. Lenaerts, M. R. van den Broeke, S. J. Déry, E. van Meijgaard, W. J. van de Berg, S. P. Palm, J. Sanz Rodrigo, Modeling drifting snow in Antarctica with a regional climate model: 1. Methods and model evaluation. *J. Geophys. Res. Atmos.* **117**, D05108 (2012).

17. C. Elkan, K. Noto, Learning classifiers from only positive and unlabeled data, in *Proceeding of the 14th ACM SIGKDD International Conference on Knowledge Discovery and Data Mining, KDD 08* (ACM, 2008), pp. 213–220.

18. K. C. Welten, L. Folco, K. Nishiizumi, M. W. Caffee, A. Grimberg, M. M. M. Meier, F. Kober, Meteoritic and bedrock constraints on the glacial history of Frontier Mountain in northern Victoria Land, Antarctica. *Earth Planet. Sci. Lett.* **270**, 308–315 (2008).

19. K. Matsuoka, A. Skoglund, G. Roth, Quantarctica (Norwegian Polar Institute, 2018); https://doi.org/10.21334/npolar.2018.8516e961 [accessed 21 August 2019].

20. K. Matsuoka, A. Skoglund, G. Roth, J. De Pomereu, H. Griffiths, R. Headland, B. Herried, K. Katsumata, A. Le Brocq, K. Licht, F. Morgan, P. D. Neff, C. Ritz, M. Scheinert, T. Tamura, A. Van De Putte, M. Van Den Broeke, A. Von Deschwanden, C. Deschamps-Berger, B. Van Liefferinge, S. Tronstad, Y. Melvær, Quantarctica, an integrated mapping environment for Antarctica, the Southern Ocean, and sub-Antarctic islands. *Environ. Model Softw.* **140**, 105015 (2021).

21. J. Mouginot, E. Rignot, B. Scheuchl, *MEaSUREs Phase-Based Antarctica Ice Velocity Map , Version 1* (NASA National Snow and Ice Data Center Distributed Active Archive Center, Boulder, Colorado USA, 2019); https://doi.org/10.5067/PZ3NJ5RXRH10 [accessed 7 August 2019].

22. K. C. Jezek, J. C. Curlander, F. Carsey, C. Wales, R. G. Barry, *RAMP AMM-1 SAR Image Mosaic of Antarctica, Version 2* (NASA National Snow and Ice Data Center Distributed Active Archive Center, Boulder, Colorado USA, 2013); https://doi.org/10.5067/8AF4ZRPULS4H [accessed 15 October 2019].

23. K. C. Jezek, Glaciological properties of the Antarctic ice sheet from RADARSAT-1 synthetic aperture radar imagery. *Ann. Glaciol.* **29**, 286–290 (1999).

24. I. C. Brown, T. A. Scambos, Satellite monitoring of blue-ice extent near Byrd Glacier, Antarctica. *Ann. Glaciol.* **39**, 223–230 (2004).

25. R. J. Arthern, D. P. Winebrenner, D. G. Vaughan, Antarctic snow accumulation mapped using polarization of 4.3-cm wavelength microwave emission. *J. Geophys. Res. Atmos.* **111**, D06107 (2006).

26. G. Baccolo, B. Delmonte, P. B. Niles, G. Cibin, E. Di Stefano, D. Hampai, L. Keller, V. Maggi, A. Marcelli, J. Michalski, C. Snead, M. Frezzotti, Jarosite formation in deep Antarctic ice provides a window into acidic, water-limited weathering on Mars. *Nat. Commun.* **12**, 436 (2021).

27. A. R. D. Smedley, G. W. Evatt, A. Mallinson, E. Harvey, Solar radiative transfer in Antarctic blue ice: Spectral considerations, subsurface enhancement, inclusions, and meteorites. *Cryosphere* **14**, 789–809 (2020).

28. G. W. Evatt, M. J. Coughlan, K. H. Joy, A. R. D. Smedley, P. J. Connolly, I. D. Abrahams, A potential hidden layer of meteorites below the ice surface of Antarctica. *Nat. Commun.* **7**, 10679 (2016).

29. Z. Wan, S. Hook, G. Hulley, *MOD11A2 MODIS/Terra Land Surface Temperature/Emissivity 8-Day L3 Global 1km SIN Grid V006* (NASA EOSDIS Land Processes DAAC, 2015); https://doi.org/10.5067/MODIS/MOD11A2.006 [accessed 13 January 2020].

30. C. H. Reijmer, J. Oerlemans, Temporal and spatial variability of the surface energy balance in Dronning Maud Land, East Antarctica. *J. Geophys. Res.* **107**, 4759 (2002).

31. S. Takahashi, T. Endoh, N. Azuma, S. Meshida, Bare ice fields developed in the inland part of Antarctica. *Proc. NIPR Symp. Polar Meteorol. Glaciol.* **5**, 128–139 (1992).

32. W. Thiery, I. V. Gorodetskaya, R. Bintanja, N. P. M. Van Lipzig, M. R. Van Den Broeke, C. H. Reijmer, P. Kuipers Munneke, Surface and snowdrift sublimation at Princess Elisabeth station, East Antarctica. *Cryosphere* **6**, 841–857 (2012).

33. I. M. Howat, C. Porter, B. E. Smith, M. J. Noh, P. Morin, The Reference Elevation Model of Antarctica. *Cryosphere* **13**, 665–674 (2019).

34. A. Burton-Johnson, M. Black, T. F. Peter, J. Kaluza-Gilbert, An automated methodology for differentiating rock from snow, clouds and sea in Antarctica from Landsat 8 imagery: A new rock outcrop map and area estimation for the entire Antarctic continent. *Cryosphere* **10**, 1665–1677 (2016).

35. J. Mouginot, E. Rignot, B. Scheuchl, Continent-wide, interferometric SAR phase, mapping of Antarctic ice velocity. *Geophys. Res. Lett.* **46**, 9710–9718 (2019).

36. M. Morlighem, *MEaSUREs BedMachine Antarctica, Version 2* (NASA National Snow and Ice Data Center Distributed Active Archive Center, Boulder, Colorado USA, 2020); https://doi.org/10.5067/E1QL9HFQ7A8M [accessed 5 October 2020].

37. W. J. Leong, H. J. Horgan, DeepBedMap: A deep neural network for resolving the bed topography of Antarctica. *Cryosphere* **14**, 3687–3705 (2020).

38. P. Fretwell, H. D. Pritchard, D. G. Vaughan, J. L. Bamber, N. E. Barrand, R. Bell, C. Bianchi, R. G. Bingham, D. D. Blankenship, G. Casassa, G. Catania, D. Callens, H. Conway, A. J. Cook, H. F. J. Corr, D. Damaske, V. Damm, F. Ferraccioli, R. Forsberg, S. Fujita, Y. Gim, P. Gogineni, J. A. Griggs, R. C. A. Hindmarsh, P. Holmlund, J. W. Holt, R. W. Jacobel, A. Jenkins, W. Jokat, T. Jordan, E. C. King, J. Kohler, W. Krabill, M. Riger-Kusk, K. A. Langley, G. Leitchenkov, C. Leuschen, B. P. Luyendyk, K. Matsuoka, J. Mouginot, F. O. Nitsche, Y. Nogi, O. A. Nost, S. V. Popov, E. Rignot, D. M. Rippin, A. Rivera, J. Roberts, N. Ross, M. J. Siegert, A. M. Smith, D. Steinhage, M. Studinger, B. Sun, B. K. Tinto, B. C. Welch, D. Wilson, D. A. Young, C. Xiangbin, A. Zirizzotti, Bedmap2: Improved ice bed, surface and thickness datasets for Antarctica. *Cryosphere* **7**, 375–393 (2013).

39. M. Morlighem, E. Rignot, T. Binder, D. Blankenship, R. Drews, G. Eagles, O. Eisen, F. Ferraccioli, R. Forsberg, P. Fretwell, V. Goel, J. S. Greenbaum, H. Gudmundsson, J. Guo, V. Helm, C. Hofstede, I. Howat, A. Humbert, W. Jokat, N. B. Karlsson, W. S. Lee, K. Matsuoka, R. Millan, J. Mouginot, J. Paden, F. Pattyn, J. Roberts, S. Rosier, A. Ruppel, H. Seroussi, E. C. Smith, D. Steinhage, B. Sun, M. R. van den Broeke, T. D. van Ommen, M. van Wessem, D. A. Young, Deep glacial troughs and stabilizing ridges unveiled beneath the margins of the Antarctic ice sheet. *Nat. Geosci.* **13**, 132–137 (2020).

40. L. Gerrish, P. Fretwell, P. Cooper, *Rock Outcrop medium resolution v7.1* (Polar Data Centre, Natural Environment Research Council, UK Research & Innovation, 2019); https://doi.org/10.5285/33d793a4-b38c-4479-90de-5ad3cec03e17 [accessed 22 November 2019].

41. H. Liu, L. Wang, K. C. Jezek, Automated delineation of dry and melt snow zones in Antarctica using active and passive microwave observations from space. *IEEE Trans. Geosci. Remote Sens.* **44**, 2152–2163 (2006).

42. P. Lee, W. Cassidy, D. Apostolopoulos, D. Bassi, L. Bravo, H. Cifuentes, M. Deans, A. Foessel, S. Moorehead, M. Parris, C. Puebla, L. Pedersen, Search for Meteorites at Martin Hills and Pirrit Hills, Antarctica. *Proceedings of Lunar and Planetary Science Conference XXX*, abstract 2046 (1999).

43. B. Choi, M. Kusakabe, Antarctic meteorites recovered from thiel mountains , west Antarctica by the First Korea expedition for Antarctic meteorites. *Meteorit. Planet. Sci. Suppl.* **42**, 5173 (2007).

44. P. Lee, W. A. Cassidy, D. Apostolopoulos, M. Deans, A. Foessel, C. Krause, J. Parra, L. Pedersen, K. Schwer, W. L. Whittaker, Search for meteorites in the Patriot Hills area, Ellsworth mountains, West Antarctica. *Meteorit. Planet. Sci.* **33**, A92–A93 (1998).

45. K. Yanai, H. Kojima, H. Naraoka, The Asuka-87 and Asuka-88 Collections of Antarctic meteorites: search, discoveries, intial processing, and preliminary identification and classification. *Proc. NIPR Symp. Antarct. Meteroites*. **6**, 137–147 (1993).

46. R. P. Harvey, Moving targets: The effect of supply, wind movement, and search losses on Antarctic meteorite size distributions, in *Workshop on Meteorites from Cold and Hot Deserts, LPI Technical Report Number 95–02*, L. Schultz, J. O. Annexstad, M. E. Zolensky, Eds. (Lunar and Planetary Institute, 1995), pp. 34–36.

47. L. Folco, K. C. Welten, A. J. T. Jull, K. Nishiizumi, A. Zeoli, Meteorites constrain the age of Antarctic ice at the Frontier Mountain blue ice field (northern Victoria Land). *Earth Planet. Sci. Lett.* **248**, 209–216 (2006).

48. M. Reichstein, G. Camps-Valls, B. Stevens, M. Jung, J. Denzler, N. Carvalhais, Prabhat, Deep learning and process understanding for data-driven Earth system science. *Nature* **566**, 195–204 (2019).

49. R. P. Harvey, A. Meibom, H. Haack, Meteorite stranding surfaces and the Greenland icesheet. *Meteorit. Planet. Sci.* **36**, 807–816 (2001).

50. C. M. Corrigan, L. C. Welzenbach, K. Righter, K. M. McBride, T. J. McCoy, R. P. Harvey, C. E. Satterwhite, A Statistical Look at the U.S. Antarctic Meteorite Collection, in *35 Seasons of the U.S. Antarctic Meteorites (1976–2010): A Pictorial Guide to the Collection*, K. Righter, C. M. Corrigan, T. J. McCoy, R. P. Harvey, Eds. (AGU and Wiley, 2014), pp. 173–187.

51. K. C. Welten, K. Nishiizumi, M. W. Caffee, D. J. Hillegonds, J. A. Johnson, A. J. T. Jull, R. Wieler, L. Folco, Terrestrial ages, pairing, and concentration mechanism of Antarctic chondrites from Frontier Mountain, Northern Victoria Land. *Meteorit. Planet. Sci.* **41**, 1081–1094 (2006).

52. B. Miao, Z. Xia, C. Zhang, R. Ou, Y. Sun, Progress of Antarctic meteorite survey and research in China. *Adv. Polar Sci.* **29**, 61–78 (2018).

53. K. Righter, J. Schutt, N. Lunning, R. Harvey, J. Karner, Identification and pairing reassessment of unequilibrated ordinary chondrites from four Antarctic dense collection areas. *Meteorit. Planet. Sci.* **56**, 1556–1573 (2021).

54. W. Li, Q. Guo, C. Elkan, A positive and unlabeled learning algorithm for one-class classification of remote-sensing data. *IEEE Trans. Geosci. Remote Sens.* **49**, 717–725 (2011).

55. G. H. John, P. Langley, Estimating Continuous Distributions in Bayesian Classifiers. *Proceedings of the Eleventh Conference on Uncertainty of Artificial Intelligence*, 338–345 (1995).

56. B. W. Silverman, *Density Estimation for Statistics and Data Analysis* (Chapman and Hall, 1986).

57. C. Schaaf, Z. Wang, *MCD43A3 MODIS/Terra+Aqua BRDF/Albedo Daily L3 Global - 500m V006* (NASA EOSDIS Land Processes DAAC, 2015); https://doi.org/10.5067/MODIS/MCD43A3.006 [accessed 23 February 2021].

58. J. Stroeve, J. E. Box, F. Gao, S. Liang, A. Nolin, C. Schaaf, Accuracy assessment of the MODIS 16-day albedo product for snow: Comparisons with Greenland in situ measurements. *Remote Sens. Environ.* **94**, 46–60 (2005).

59. B. Noël, C. L. Jakobs, W. J. J. van Pelt, S. Lhermitte, B. Wouters, J. Kohler, J. O. Hagen, B. Luks, C. H. Reijmer, W. J. van de Berg, M. R. van den Broeke, Low elevation of Svalbard glaciers drives high mass loss variability. *Nat. Commun.* **11**, 4597 (2020).

60. N. Imae, V. Debaille, Y. Akada, W. Debouge, S. Goderis, G. Hublet, T. Mikouchi, N. Van Roosbroek, A. Yamaguchi, H. Zekollari, P. Claeys, H. Kojima, Report of the JARE-54 and BELARE 2012-2013 joint expedition to collect meteorites on the Nansen Ice Field, Antarctica. *Antarct. Rec.* **59**, 38–72 (2015).

61. R. Bindschadler, P. Vornberger, A. Fleming, A. Fox, J. Mullins, D. Binnie, S. J. Paulsen, B. Granneman, D. Gorodetzky, The landsat image mosaic of Antarctica. *Remote Sens. Environ.* **112**, 4214–4226 (2008).

62. S. Goderis, M. Yesiltas, H. Pourkhorsandi, N. Shirai, M. Poudelet, M. Leitl, A. Yamaguchi, V. Debaille, P. Claeys, A detailed record of the BELARE 2019-2020 meteorite recovery expedition on the Nansen Ice Field, East Antarctica. *Antarct. Rec.* **65**, 1–20 (2021).

63. W. A. Cassidy, Antarctic search for meteorites. *Antarct. J. U.S.* **XII**, 96–98 (1977).

64. National Aeronautics and Space Administration, Antarctic Meteorites Recovery Locations Map (2017); https://curator.jsc.nasa.gov/antmet/map.cfm [accessed 26 May 2021].

65. L. Martel, Searching Antarctic Ice for Meteorites. *Planet. Sci. Res. Discov.* (2002); www.psrd.hawaii.edu/Feb02/meteoriteSearch.html [accessed 26 January 2020].

66. K. Yanai, K. Shiraishi, H. Kojima, The Asuka-90 meteorites collection from Antarctica: searching, initial processing and preliminary identification. *Proc. NIPR Symp. Antarct. Meteorites.* **7**, 1–8 (1994).

67. P. Claeys, N. Mattielli, V. Debaille, S. Goderis, Search of Antarctic meteorites: Belgian Activities "SAMBA", final report. *Belgian Sci. Policy* (2015).

68. J. Schutt, L. Schultz, E. Zinner, M. Zolensky, Search for meteorites in the Allan Hills region, 1985-1986. *Antarct. J. U.S.* **XXI**, 82–83 (1986).

69. W. A. Cassidy, J. Schutt, Antarctic search for meteorites: Field proram 1984-1985. *Antarct. J. U.S.* **XIX**, 54 (1985).

70. R. P. Harvey, J. W. Schutt, Meteorite recovery and reconnaissance near Pecora Escarpment and surrounding regions. *Antarct. J. U.S.* **XXVII**, 26–28 (1992).

71. U. B. Marvin, The field season in Victoria Land, 1978-1979. *Smithson. Contrib. Earth Sci.* **24**, 3–8 (1982).

72. W. A. Cassidy, The 1980-1981 field season. *Smithson. Contrib. Earth Sci.* **26**, 5–8 (1984).

73. F. Coren, G. Delisle, P. Sterzai, Ice dynamics of the Allan Hills meteorite concentration sites revealed by satellite aperture radar interferometry. *Meteorit. Planet. Sci.* **38**, 1319–1330 (2003).

74. W. A. Cassidy, J. O. Annexstad, Antarctic search for meteorites, 1980-1981. *Antarct. J. U.S.* **XVI**, 61–62 (1981).

75. W. A. Cassidy, Antarctic search for meteorites (ANSMET 1978-79). *Antarct. J. U.S.* **XIV**, 41–42 (1979).

76. U. B. Marvin, The origin and early history of the U.S. Antarctic Search for Meteorites Program (ANSMET), in *35 Seasons of the U.S. Antarctic Meteorites (1976–2010): A Pictorial Guide to the Collection*, K. Righter, C. M. Corrigan, T. J. McCoy, R. P. Harvey, Eds. (AGU and Wiley, 2014), pp. 1–22.

77. R. P. Harvey, J. W. Schutt, Meteorite recovery and reconnaissance in the Allan Hills-David Glacier region, 1992-1993. *Antarct. J. U.S.* **XXVIII**, 51–52 (1993).

78. J. Schutt, Results of the antarctic search for meteorites, 1981-1982. *Antarct. J. U.S.* **XVII**, 56–57 (1982).

79. G. R. Huss, J. Wagstaff, P. J. Wasilewski, C. Thompson, Search for meteorites north and west of Elephant Moraine, Victoria Land, 1987-1988. *Antarct. J. U.S.* **XXIII**, 47–49 (1988).

80. W. A. Cassidy, Field occurrences and collecting procedures. *Smithson. Contrib. Earth Sci.* **23**, 3–7 (1980).

81. J. Schutt, L. A. Rancitelli, U. Krähenbühl, R. Crane, Exploration for meteorite concentrations in the Thiel Mountains/Pecora Escarpment reion, 1982-1983. *Antarct. J. U.S.* **XVIII**, 83–86 (1983).

82. J. W. Schutt, The expedition to the thiel mountains and Pecora escarpment, 1982-1983. *Smithson. Contrib. Earth Sci.* **28**, 10–15 (1989).

83. W. A. Cassidy, Antarctic search for meteorites during the 1977-78 field season. *Antarct. J. U.S.* **XIII**, 39–40 (1978).

84. W. A. Cassidy, Meteorite search at Lewis Cliff ice tongue: Systematic recovery program completed. *Antarct. J. U.S.* **XXIV**, 44 (1989).

85. A. Grinsted, J. Moore, V. B. Spikes, A. Sinisalo, Dating Antarctic blue ice areas using a novel ice flow model. *Geophys. Res. Lett.* **30**, 1–5 (2003).

86. R. P. Harvey, J. W. Schutt, Meteorite recovery and reconnaissance in the Allan Hills-David Glacier and Darwin Glacier regions , 1996-1997. *Antarct. J. U.S.* **XXXII**, 25–27 (1997).

87. G. Delisle, I. Frachi, A. Rossi, R. Wieler, Meteorite finds by EUROMET near Frontier Mountain, North Victoria Land, Antarctica. *Meteoritics* **28**, 126–129 (1993).
